# A Hybrid Metaheuristic Based on Neurocomputing for Analysis of Unipolar Electrohydrodynamic Pump Flow

**DOI:** 10.3390/e23111513

**Published:** 2021-11-14

**Authors:** Muhammad Fawad Khan, Muhammad Sulaiman, Carlos Andrés Tavera Romero, Ali Alkhathlan

**Affiliations:** 1 Department of Mathematics, Abdul Wali Khan University, Mardan 23200, Pakistan; fawadaurang@gmail.com; 2 COMBA R&D Laboratory, Faculty of Engineering, Universidad Santiago de Cali, Cali 76001, Colombia; carlos.tavera00@usc.edu.co; 3 Computer Science Department, Faculty of Computing and Information Technology, King Abdulaziz University, Jeddah 21589, Saudi Arabia; analkhathlan@kau.edu.sa

**Keywords:** unipolar pump flow, electrohydrodynamic, nonlinear systems, sine–cosine algorithm, sequential quadratic programming, dynamic parameters, electric potential, neurocomputing, charge density

## Abstract

A unipolar electrohydrodynamic (UP-EHD) pump flow is studied with known electric potential at the emitter and zero electric potential at the collector. The model is designed for electric potential, charge density, and electric field. The dimensionless parameters, namely the electrical source number $\left(E_{s}\right)$, the electrical Reynolds number $\left(R_{e_{E}}\right)$, and electrical slip number $\left(E_{sl}\right)$, are considered with wide ranges of variation to analyze the UP-EHD pump flow. To interpret the pump flow of the UP-EHD model, a hybrid metaheuristic solver is designed, consisting of the recently developed technique sine–cosine algorithm (SCA) and sequential quadratic programming (SQP) under the influence of an artificial neural network. The method is abbreviated as ANN-SCA-SQP. The superiority of the technique is shown by comparing the solution with reference solutions. For a large data set, the technique is executed for one hundred independent experiments. The performance is evaluated through performance operators and convergence plots.

## 1. Introduction

The combined study of electrodynamics and hydrodynamics is known as electrohydrodynamics (EHD). It has numerous applications, due to which researchers pay attention to EHD, especially in engineering. In EHD, many developments are made, especially theoretical developments to study the flow mechanism of fluid affected by an external electric field. In this mechanism, the fluid is discussed under the influence of an external electric field. That external electric field is produced due to the potential difference between collector and emitter, which applies columbic force on charged particles in the fluid. EHD has many applications such as fabrication of drugs delivery systems [1], microelectromechanical devices [2], EA transformation, investigating heat transfer [3], boundary control in hypersonic flows [4], measurement of heat transfer [5], inkjet mechanism [6], printing based on EHD [7], combustion controls [8], ethylcellulose, cellulose acetate and carboxymethyl cellulose microstructure prepared by EHD [9], untethered robots based on EHD [10], dielectric pump designs [11], embryo transport in uterine [12], and protein biomolecule separation using EHD [13], etc.

The numerical analysis, based on theoretical perspectives of EHD ion flow, was addressed by Syed et al. [14]. Relevant work was presented by Syed [15] with consideration of ion drag, pumping, and conduction. The work addressed the creation of electric fields and discusses how pumping is efficient by variation in electric field Reynold number. While [16] reported results of flow velocities and flow reliability in EHD for various fluids. The electrodes, which produce a 3D electric field, were numerically studied by Pearson in [17], and a hybrid method was studied by McKee [18] for solving an EHD flow problem in a cylindrical conduit based on Runge–Kutta, finite difference, and continuation. The existence of unique solution of reduced EHD Navier–Stokes was reported by Paullet in [19] and a network thermodynamic solver-based study was conducted in [20] for variation in flow about electric source or electric slip and Reynolds number [20].

The UP-EHD problem was solved by Beg et al. [20] using the network simulation method (NSM) with implementation of Pspice 6.0 code. The NSM with Pspice has many restrictions. This type of NSM is restricted to circuit consist of only ten resistors. The Pspice NSM does not have a supporting iterative method [21]. For the iteration process, it needs to adopt another method such as Newton–Raphson and others. If the NSM network has triangular and rectangular geometry, it has disadvantages that the controlled source, capacitor, and resistors involved in the NSM must be calculated by hand [22]. NSM Pspice has a stability problem. If three sectors are used in a circuit, one of them will be unstable. In such a situation, small time steps have to choose, which leads to the unacceptably large computational time with accumulation of rounding error [23].

The same problem was solved by Jadoon et al. [24] using the finite difference method (FDM) integrating with basic genetic algorithm (GA). The GA has many drawbacks that effect the solution of a problem. A few of limitations of the technique used in [24] are: has consistency, dependency or correlation, and distance measures. A major limitation of the methods is that they examine each feature independently and ignore the individual performance of the feature in relation to the group. It may have a combined effect on the result of techniques [25,26,27].

In recent years, numerous developments have been introduced in computational solvers, especially stochastic methods for the solution of boundary value problems [28,29,30,31,32,33,34,35,36,37,38,39,40,41,42,43], but still, the stochastic computational methods are to be implemented to analyze the dynamic behavior of electrohydrodynamics problems. The development in stochastic computational solvers, attracts the attention of researchers, exploitation with global search and hybridize with reliable local search, which provides a reliable, efficient and better convergence rate for solution of problems arising in many fields [44,45,46,47,48]. The solutions of such hybrid techniques are comparatively reliable.

A few relevant solutions of hydro- and electrohydrodynamics problems are 3D numerical microcooling analysis for an electrohydrodynamic micropump [49], neurocomputing networks for entropy generation under the influence of MHD and thermal radiation [32], electrohydrodynamic atomization process for production of polymeric composite microspheres [50], a feedforward neural network fuzzy grey predictor-based controller for force control of an electrohydraulic actuator [51], a fast convergent semianalytic method for an electrohydrodynamic flow in a circular cylindrical conduit [28], numerical analysis of electrohydrodynamic (EHD) instability in dielectric liquid–gas flows subjected to unipolar injection [30], numerical analysis of electrohydrodynamic flows of a dielectric liquid [43], and numerical investigation of electrohydrodynamic forced convection heat [31], and many other examples of EHD-based problems are available in the literature.

Many significant algorithms are proposed recently, but still, a question arises: why there is a need for more optimization techniques? The answer to this question can be found in the no free lunch (NFL) theorem [52]. The NFL theorem proves that no optimization technique can outperform all optimization problems. By solving a set of optimization problems, a technique cannot guarantee the solution of all optimization problems having different types and nature. Simply, despite the superior performance on a subset of optimization problems, the optimization techniques perform equally average for all optimization problems. The prove of NFL theorem motivate researcher to design new optimization algorithms or improve/modify the existing algorithms. This is also the motivation of this work to improve the quality of SCA by hybridization with SQP. Some of the hybridizations of SCA with SQP reported in literature are detailed as follows. Babar et al. solved the electrical dispatch problem [53], and recently, a hybrid sine cosine algorithm with SQP for solving convex, for plate-fin heat exchanger SCA was implemented by [54]. A nonconvex economic dispatch problem was reported in [55]. Similarly, for the solution of UP-EHD, the proposed scheme is a contribution to the neural-network-based solvers. The proposed methodology is implemented to investigate a reliable and robust solution for unipolar electrohydrodynamic (UP-EHD) pump flow model. The hybridization of this algorithm is simple but effective with a less mathematical formulation. The silent features of the scheme, based on the sine–cosine algorithm assisted by sequential quadratic programming, are as follows:

- In this study, a new design numerical solver is proposed for the solutions of fluid mechanics problem, unipolar EHD model, with help of hybridization of supervised and unsupervised methods, i.e., global search technique sine–cosine algorithm (SCA) and local search technique sequential quadratic programming.
- The precision of the proposed mechanism is analyzed by comparison of solution with Runge–Kutta order four (RK4) technique for each case of the model.
- Reliability, convergence, and validity of the proposed methodology, ANN-SCA-SQP algorithm, assessed through statistical analysis. Interpreted numerically by utilizing mean square error, error in Nash–Sutcliffe efficiency and root-mean-square error. Graphically analyze using convergence plots such as histogram with normal distribution and box plots.
- The designed methodology provides accurate, reliable, valid and robust solutions with defined input grids and promising convergence.

The rest of the paper is organized as follows. In Section 2, we present the mathematical model of UP-EHD pump flow. In Section 3, we introduce and discuss the proposed scheme, performance operators, and their global versions. In Section 2, we present empirical simulation and dynamic characteristics of the pump flow model based on its physical parameters. The work is concluded in Section 5.

## 2. Dynamic Model of UP-EHD Pump Flow

**Figure 1:**
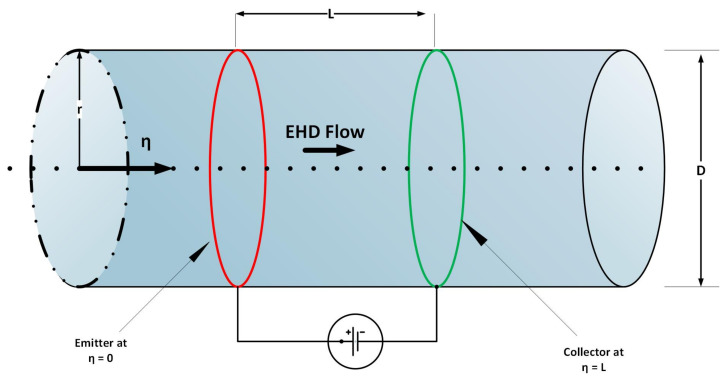
Physical structure of UP-EHD flow model.

The UP-EHD pump flow model [15,20] based on direct current (DC) consists of first-order ordinary differential equations. The physical structure of UP-EHD pump flow model is shown in Figure 1. For formulation of mathematical model of UP-EHD, the three conservation laws for an electrical field, current density, and electrical potential can be stated thus:

Gauss’s Law:

(1) $$\nabla \cdot \varepsilon \vec{E}=\rho ,$$

Conservation of charge:

(2) $$\nabla \cdot \vec{J}+\frac{\partial \rho }{\partial t}=0,$$

Conservation of electric potential:

(3) $$\vec{E}=-\nabla \Phi ,$$

Combination of all three mechanisms of the current flow leads to the following relation for total current density:

(4) $$\vec{J}=\sigma \vec{E}+\rho \vec{u}+\rho \mu \vec{E},$$

where ∇ is the gradient operator, $\vec{E}$ is electrical field vector $\left(V/m^{2}\right),\vec{J}$ is the current density vector $\left(\mathrm{Amps}/m^{2}\right),\rho$ is charge density (Coulomb$/m^{3}),t$ is time, ε is permittivity (Faradays/m), Φ is potential (V),σ is electrical conductivity $\left(S/m\right),\vec{u}$ is fluid bulk velocity vector (m/s) and μ is ion mobility $\left(m^{2}/\mathrm{Vs}\right)$. In Equation (4) the right hand side component terms designate electrical conduction, electrical convection, and ionic mobility, respectively. Herein, we shall study the case where electrical field located at emitter (i.e., at η=0 in Figure 1), is zero (maximum pressure scenario).

We consider the case where the electrical potential at emitter (z=0) is known and electrical potential at the collector (z=L) is zero (operating condition scenario). The conservation Equations (1)–(4) can be shown to reduce for constant property electrohydrodynamic flow to the following sets of ordinary differential equations, where η, i.e., axial coordinate along the axis of the DC pump, is the only independent variable:

(5) $$\left(1+E_{sl}E\left(\eta \right)\right)\rho^{\prime }\left(\eta \right)+\frac{\rho (\eta )}{R_{e_{E}}}+E_{s}E_{sl}\rho^{2}\left(\eta \right)=0,$$

(6) $$E^{\prime }\left(\eta \right)-E_{s}\rho \left(\eta \right)=0,$$

(7) $$E+\phi^{\prime }\left(\eta \right)=0,$$

the associated conditions at emitter (η=0) are:

(8) ρ(0)=1,E(0)=0,ϕ(0)=1.

Moreover, the purpose of this study is to address the dynamic characteristics of UP-EHD pump model, given in (5)–(8), with the help of a soft computing technique. For the considered case the characteristic of UP-EHD will be discussed based on the variation of electric slip number ($E_{sl}$), electric source number ($E_{s}$) and electric Reynolds number ($R_{e_{E}}$). More details about UP-EHD model are given in [15,20].

## 3. Proposed Scheme

**Figure 2:**
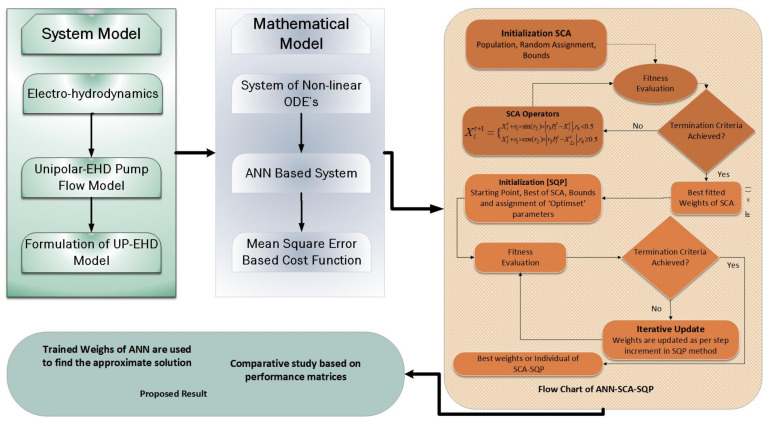
Work flow chartof proposed methodology. Initially, population in SCA is set for generation of solutions, fitness of generated solution is evaluated by SCA. The fittest solution is provided as an initial point to SQP and SQP provides the best solution as weights of ANN.

The proposed scheme is developed in two phases; the first phase discusses the construction of artificial neural network-based model, and the second phase consists of proposed optimization scheme. The structure of the scheme is shown in Figure 2.

### 3.1. Mathematical Modelling for UP-EHD

The designed mathematical modeling for UP-EHD has two parts. In the first part, the strength of Artificial neural networks is implemented for development of mathematical model and the second part consist of construction of a fitness function for the UP-EHD model based on mean square errors. The ANNs based model is as follows:

(9) $$\left.\begin{array}{c} \hat{\rho }\left(\eta \right)=\sum_{i=1}^{k}a_{\rho i}\theta \left(w_{\rho i}\left(\eta \right)+b_{\rho i}\right)\\ {\hat{\rho }}^{\prime }\left(\eta \right)=\sum_{i=1}^{k}a_{\rho i}\theta^{\prime }\left(w_{\rho i}\left(\eta \right)+b_{\rho i}\right)\\ {\hat{\rho }}^{\prime \prime }\left(\eta \right)=\sum_{i=1}^{k}a_{\rho i}\theta^{\prime \prime }\left(w_{\rho i}\left(\eta \right)+b_{\rho i}\right)\\ \vdots \\ {\hat{\rho }}^{\left(n\right)}\left(\eta \right)=\sum_{i=1}^{k}a_{\rho i}\theta^{\left(n\right)}\left(w_{\rho i}\left(\eta \right)+b_{\rho i}\right) \end{array}\right\},$$

(10) $$\left.\begin{array}{c} \hat{E}\left(\eta \right)=\sum_{i=1}^{k}a_{Ei}\theta \left(w_{Ei}\left(\eta \right)+b_{Ei}\right)\\ {\hat{E}}^{\prime }\left(\eta \right)=\sum_{i=1}^{k}a_{Ei}\theta^{\prime }\left(w_{Ei}\left(\eta \right)+b_{Ei}\right)\\ {\hat{E}}^{\prime \prime }\left(\eta \right)=\sum_{i=1}^{k}a_{Ei}\theta^{\prime \prime }\left(w_{Ei}\left(\eta \right)+b_{Ei}\right)\\ \vdots \\ {\hat{E}}^{\left(n\right)}\left(\eta \right)=\sum_{i=1}^{k}a_{Ei}\theta^{\left(n\right)}\left(w_{Ei}\left(\eta \right)+b_{Ei}\right) \end{array}\right\},$$

(11) $$\left.\begin{array}{c} \hat{\phi }\left(\eta \right)=\sum_{i=1}^{k}a_{\phi i}\theta \left(w_{\phi i}\left(\eta \right)+b_{\phi i}\right)\\ {\hat{\phi }}^{\prime }\left(\eta \right)=\sum_{i=1}^{k}a_{\phi i}\theta^{\prime }\left(w_{\phi i}\left(\eta \right)+b_{\phi i}\right)\\ {\hat{\phi }}^{\prime \prime }\left(\eta \right)=\sum_{i=1}^{k}a_{\phi i}\theta^{\prime \prime }\left(w_{\phi i}\left(\eta \right)+b_{\phi i}\right)\\ \vdots \\ {\hat{\phi }}^{\left(n\right)}\left(\eta \right)=\sum_{i=1}^{k}a_{\phi i}\theta^{\left(n\right)}\left(w_{\phi i}\left(\eta \right)+b_{\phi i}\right) \end{array}\right\},$$

**Figure 3:**
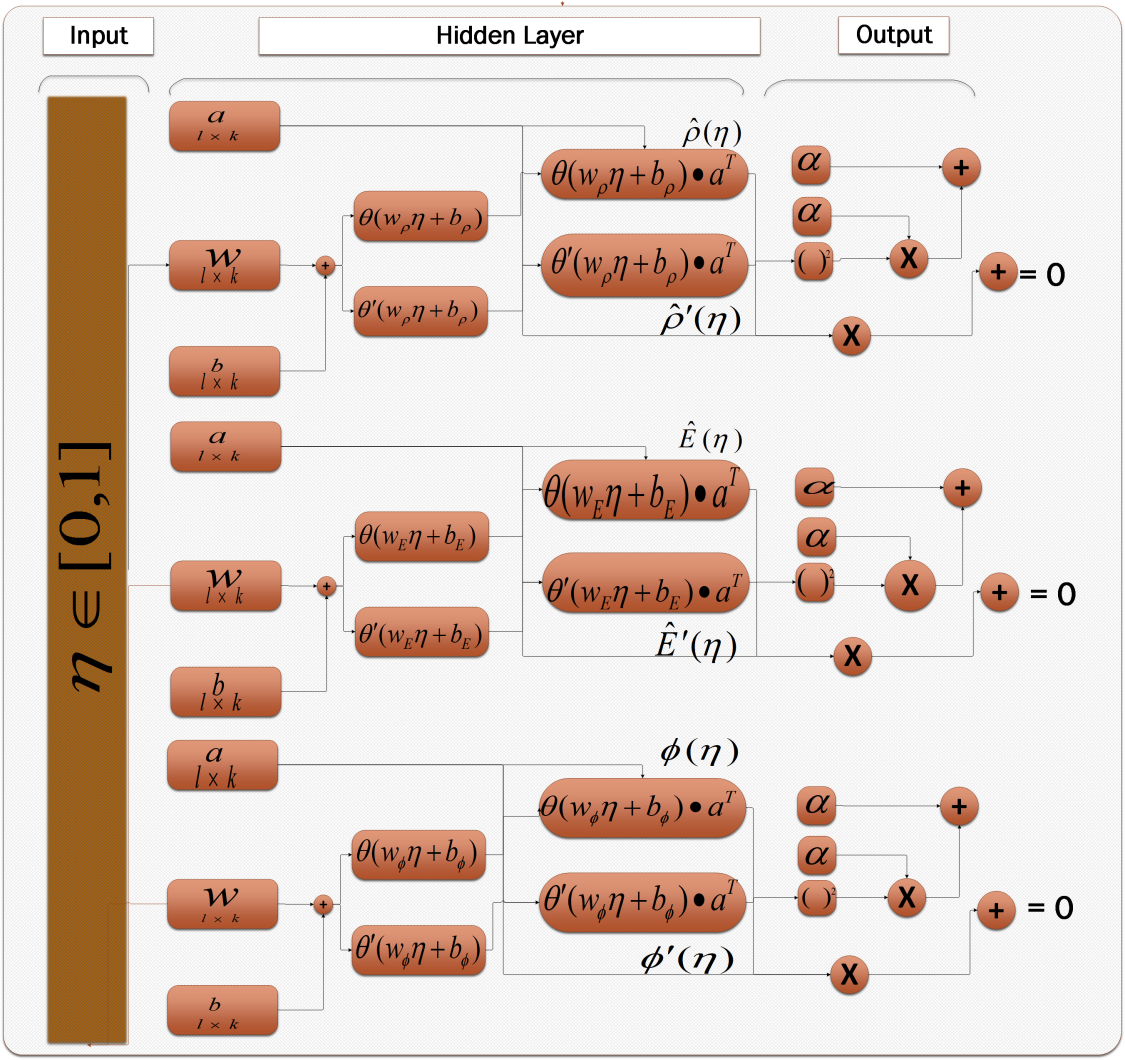
The architecture of ANN model. With input points. It’s the simplest form of ANN. A unidirectional network with no cycle has three layers input, hidden, and output layers. In inputs, η∈[0,1] is taken for an initial guess of unknown weights. For the hidden layer, sigmoid function is used as given in Equation (12).

Equations (9)–(11) are ANNs based model for the solutions of ρ,E and ϕ and their respective derivatives. Learning process of ANN model is given in Figure 3.

The equations are constructed by using activation function in Equation (12) and its ANN form is given in Equations (13) and (14).

(12) $$\theta \left(x\right)=\frac{1}{1+e^{-x}},$$

(13) $$\hat{\theta }\left(\eta \right)=\sum_{i=1}^{k}a_{i}\left(\frac{1}{1+e^{-\left(w_{i}\eta +b_{i}\right)}}\right),$$

(14) $${\hat{\theta }}^{\prime }\left(\eta \right)=\sum_{i=1}^{k}a_{i}w_{i}\left(\frac{e^{-\left(w_{i}\eta +b_{i}\right)}}{{\left(1+e^{-\left(w_{i}\eta +b_{i}\right)}\right)}^{2}}\right),$$

Equation (13) based on set W of variables called weights as $W=\left[a_{i},w_{i},b_{i}\right],\mathrm{where}\;a_{i}=\left[a_{1},a_{2},\ldots a_{k}\right],w_{i}=\left[w_{1},w_{2},\ldots w_{k}\right]$ and $b_{i}=\left[b_{1},b_{2},\ldots b_{k}\right]$.

Now, the fitness function for the UP-EHD system, using unsupervised errors, is constructed by least-square errors. Least squares problems arise in the context of fitting a parameterized mathematical model to a set of data points by minimizing an objective expressed as the sum of the squares of the errors between the model function and a set of data points. If a model is linear in its parameters, the least squares objective is quadratic in the parameters. The least-square method is to identify the best weights for the solution of the system by minimizing the least square errors in every single equation of a system [56,57]. Least square method can estimate and remove the correlated errors in the problems [58]. It is the routine method for solution of problems that consists of differential equations. The least square method need an iterative technique for which ANN-SCA-SQP algorithm is used for minimization of error. It was used for solution of problems in heat transfer and fluid mechanics such as [29,59,60,61]. The cost function in term of error is given as:

(15) $$min\;e=e_{\rho }+e_{E}+e_{\phi }+e_{C},$$

where $e_{\rho }$, $e_{E}$ and $e_{\phi }$ are error functions associated with the equations of the system (5)–(7) respectively as:

(16) $$\begin{array}{c} e_{\rho }=\frac{1}{N}\sum_{m=1}^{N}{\left(\left(1+E_{sl}{\hat{E}}_{m}\right){\hat{\rho }}_{m}^{\prime }\left(\eta \right)+\frac{{\hat{\rho }}_{m}}{R_{e_{E}}}+E_{s}E_{sl}{\hat{\rho }}_{m}^{2}\right)}^{2},\\ \;\;\;\eta \in (0,1), \end{array}$$

(17) $$e_{E}=\frac{1}{N}\sum_{m=1}^{N}{\left({\hat{E}}^{\prime }\left(\eta \right)-E_{s}\hat{\rho }\right)}^{2},\;\eta \in \left(0,1\right),$$

(18) $$e_{\phi }=\frac{1}{N}\sum_{m=1}^{N}{\left(E+{\hat{\phi }}^{\prime }\left(\eta \right)\right)}^{2},\;\eta \in \left(0,1\right),$$

here $N=\frac{1}{h}$, *h* is step size in a given span. And $e_{C}$ is error function associated with initial conditions as:

(19) $$e_{C}=\frac{1}{3}\left({\left(\hat{\rho }-1\right)}^{2}+{\hat{E}}^{2}+{\left(\hat{\phi }-1\right)}^{2}\right).$$

### 3.2. Optimization Procedure

To analyze the dynamic behavior of the UP-EHD model, the fitness function defined in Equation (15) is optimized with a global search method based on SCA hybridize with SQP for local refinements. A brief description, in form of a flow chart of SCA-SQP, is given in Figure 2. The SCA was first presented by Syedali [62]. The algorithm is population-based. Generally, the population based algorithms generate multiple random initial solutions and further converge toward the required best solution. The mathematical theory of SCA is based on sine and cosine trigonometric functions. The following equations are used in SCA for updating the position of solutions:

(20) $$\begin{array}{c} X_{i}^{t+1}=X_{i}^{t}+r_{1}\times \sin\left(r_{2}\right)\times \left|r_{3}P_{i}^{t}-X_{i}^{t}\right|,\\ X_{i}^{t+1}=X_{i}^{t}+r_{1}\times \cos\left(r_{2}\right)\times \left|r_{3}P_{l}^{t}-X_{i_{j}}^{t}\right|, \end{array}$$

where, the position of current solution in $i^{th}$ dimension at $t^{th}$ iteration is denoted by $X_{i}^{t}$, $r_{1}/r_{2}/r_{3}$ are random numbers, $P_{i}$ indicate the position of destination point and || denote absolute value.

The Equation (20) with parametric values can be written as:

(21) $$X_{l}^{t+1}=\left\{\begin{array}{cc} X_{i}^{t}+r_{1}\times \sin\left(r_{2}\right)\times \left|r_{3}P_{l}^{t}-X_{i}^{t}\right|, & r_{4}<0.5,\\ X_{l}^{t}+r_{1}\times \cos\left(r_{2}\right)\times \left|r_{3}P_{l}^{t}-X_{L_{i}}^{t}\right|, & r_{4}\geq 0.5. \end{array}\right.$$

where $r_{4}$ indicate a random value in [0, 1]. In the above equation, there are four parameters $r_{1},r_{2},r_{3}$, and $r_{4}$. The $r_{1}$ indicates the movement direction which could be feasible or outside that region. Parameter $r_{2}$ describes the distance from the region that how far the direction is toward or outward the target. Parameter $r_{3}$ defines weights for target value and $r_{4}$ equally operate sine and cosine constituent of Equation (21). The SCA is a reliable and consistent technique, implemented for many problems and found promising. SCA has much application such as designing bend photonic crystal [63], hydro-thermal-wind scheduling [64], construction duration and schedule robustness [65], wind plant energy production [66], automatic voltage regulator system set up [67], etc. For further improvement, in this study, the SCA is hybridized with sequential Quadratic programming (SQP) under the influence of artificial neural network. SQP is a fast converging local search technique [68]. Sequential quadratic programming is a type of technique that can be implemented for quadratic problems and nonlinear real world problems. It has much application and can solve numerous problems such as nonlinear least squares estimation [69], control allocation with singularity avoidance [70], distributing optical parameters depend on the time-domain radiative [71], control of building HVAC and R systems [72], Optimality of a rod-shape ultrasonic motor [73] and computational intelligence paradigm for nonlinear electric circuit models [74], etc. Hybridization of SCA with fast converging local search technique improve the convergence of SCA. The proposed mechanism throughout the study is abbreviated as ANN-SCA-SQP. The performance of the proposed scheme is found consistent and reliable. For the validity of the scheme, the solutions are graphically tallied with Rang-Kutta order 4 (RK4). The overlapping of solutions of the UP-EHD model obtained through ANN-SCA-SQP algorithm with solutions of RK4 verify convergence of ANN-SCA-SQP algorithm. In this hybrid technique, the global search algorithm SCA explores and local search technique SQP refine and improve the quality of the solution of the nonlinear UP-EHD pump flow model.

The flow chart in blocks structure of ANN-SCA-SQP algorithm is given in Figure 2, which illustrates mechanism and structure of proposed scheme.

### 3.3. Performance Operators

Statistical performance operators are used for the evaluation of proposed scheme, i.e., ANN-SCA-SQP algorithm. The performance indices are Mean Absolute Deviation (MAD), Root Mean Squared Error (RMSE) and Error in Nash-Sutcliffe Efficiency (ENSE) based on Nash-Sutcliffe Efficiency (NSE). Mathematical definitions of the mentioned operators for the solution of UP-EHD, in terms of ρ, E, and ϕ, are as given:

(22) $$\left[\begin{array}{c} {\mathrm{MAD}}_{\rho }\;=\frac{1}{\;m}\sum_{i=1}^{m}\left|\rho_{i}-{\hat{\rho }}_{i}\right|\\ {\mathrm{MAD}}_{E}\;=\;\frac{1}{\;m}\sum_{i=1}^{m}\left|E_{i}-{\hat{E}}_{i}\right|\\ {\mathrm{MAD}}_{\phi }\;=\frac{1}{\;m}\sum_{i=1}^{m}\left|\phi_{i}-{\hat{\phi }}_{i}\right| \end{array}\right],$$

(23) $$\left[\begin{array}{c} {\mathrm{RMSE}}_{\rho }\;=\sqrt{\frac{1}{m}\sum_{i=1}^{m}{\left(\rho_{i}-{\hat{\rho }}_{i}\right)}^{2}}\\ {\mathrm{RMSE}}_{E}\;=\sqrt{\frac{1}{m}\sum_{i=1}^{m}{\left(E_{i}-{\hat{E}}_{i}\right)}^{2}}\\ {\mathrm{RMSE}}_{\phi }\;=\sqrt{\frac{1}{m}\sum_{i=1}^{m}{\left(\phi_{i}-{\hat{\phi }}_{i}\right)}^{2}}, \end{array}\right],$$

(24) $$\left[\begin{array}{c} {\mathrm{NSE}}_{\rho }\;=1-\frac{\sum_{i=1}^{m}{\left(\rho_{i}-{\hat{\rho }}_{i}\right)}^{2}}{\sum_{i=1}^{m}{\left(\rho_{i}-{\bar{\rho }}_{i}\right)}^{2}}\\ {\mathrm{NSE}}_{E}\;=1-\frac{\sum_{i=1}^{m}{\left(E_{i}-{\hat{E}}_{i}\right)}^{2}}{\sum_{i=1}^{m}{\left(E_{i}-{\bar{E}}_{i}\right)}^{2}}\\ {\mathrm{NSE}}_{\phi }\;=1-\frac{\sum_{i=1}^{m}{\left(\phi_{i}-{\hat{\phi }}_{i}\right)}^{2}}{\sum_{i=1}^{m}{\left(\phi_{i}-{\bar{\phi }}_{i}\right)}^{2}}, \end{array}\right],$$

(25) $$\begin{array}{c} \left[\begin{array}{c} \left[ENSE_{\rho }\;ENSE_{E}\;ENSE_{\phi }\right]\\ =\left[\left|1-NSE_{\rho }\right|\right.\;\left|1-NSE_{E}\right|\;\left.\left|1-NSE_{\phi }\right|\right] \end{array}\right]. \end{array}$$

Here *m* is number of input points, while ρ, *E*, and ϕ are the empirical reference solutions obtained through the Runge–Kutta order four (RK4) method, ρ, *E*, and ϕ are their average values, while $\hat{\rho }$, $\hat{E}$, and $\hat{\phi }$, are approximated solutions by proposed scheme.

The suitable or desire value of performance evaluators MAD, RMSE and ENSE is zero. While the value of NSE is 1. The ENSE operator is based on NSE. To check the reliability, effectiveness and efficiency of proposed methodology, a global version of the above operators is also used. The global versions of discussed performance indices are named: MAD as GMAD, RMSE as GRMSE and ENSE as GENSE. Multiple runs are executed for collection of a large data set. The mathematical formulation of the global performance indices are given as:

(26) $$\left[\begin{array}{c} {\mathrm{GMAD}}_{\rho }\;=\frac{1}{I}\sum_{i=1}^{I}{\left(\frac{1}{m}\sum_{k=1}^{m}\left(\left|\rho_{i}-{\hat{\rho }}_{i}\right|\right)\right)}_{i}\\ {\mathrm{GMAD}}_{E}\;=\frac{1}{I}\sum_{i=1}^{I}{\left(\frac{1}{m}\sum_{k=1}^{m}\left(\left|E_{i}-{\hat{E}}_{i}\right|\right)\right)}_{i}\\ GMAD_{\phi }\;=\frac{1}{I}\sum_{i=1}^{I}{\left(\frac{1}{m}\sum_{K=1}^{m}\left(\left|\phi_{i}-{\hat{\phi }}_{i}\right|\right)\right)}_{i} \end{array}\right],$$

(27) $$\left[\begin{array}{c} {\mathrm{GRMSE}}_{\rho }\;=\frac{1}{I}\sum_{i=1}^{I}{\left(\sqrt{\frac{1}{m}\sum_{k=1}^{m}{\left(\rho_{i}-{\hat{\rho }}_{i}\right)}^{2}}\right)}_{i}\\ {\mathrm{GRMSE}}_{E}\;=\frac{1}{I}\sum_{i=1}^{I}{\left(\sqrt{\frac{1}{m}\sum_{k=1}^{m}{\left(E_{i}-{\hat{E}}_{i}\right)}^{2}}\right)}_{i}\\ {\mathrm{GRMSE}}_{\phi }\;=\frac{1}{I}\sum_{i=1}^{I}{\left(\sqrt{\frac{1}{m}\sum_{k=1}^{m}{\left(\phi_{i}-{\hat{\phi }}_{i}\right)}^{2}}\right)}_{i} \end{array}\right],$$

(28) $$\left[\begin{array}{c} {\mathrm{GENSE}}_{\rho }\;=\frac{1}{I}\sum_{i=1}^{I}{\left(1-\frac{\sum_{k=1}^{m}{\left(\rho_{i}-{\hat{\rho }}_{i}\right)}^{2}}{\sum_{k=1}^{m}{\left(\rho_{i}-{\hat{\rho }}_{i}\right)}^{2}}\right)}_{i}\\ {\mathrm{GENSE}}_{E}\;==\;\frac{1}{I}\sum_{i=1}^{I}{\left(1-\frac{\sum_{k=1}^{m}{\left(E_{i}-{\hat{E}}_{i}\right)}^{2}}{\sum_{k=1}^{m}{\left(E_{i}-{\hat{E}}_{i}\right)}^{2}}\right)}_{i}\\ {\mathrm{GENSE}}_{\phi }\;=\frac{1}{I}\sum_{i=1}^{I}{\left(1-\frac{\sum_{k=1}^{m}{\left(\phi_{i}-{\hat{\phi }}_{i}\right)}^{2}}{\sum_{k=1}^{m}{\left(\phi_{i}-{\hat{\phi }}_{i}\right)}^{2}}\right)}_{i} \end{array}\right],$$

In Equations (26)–(28), *I* represent number of runs. The proposed scheme is executed for 100 independent runs. All three global operators depend on the average values of their respective operators.

## 4. Empirical Simulation

**Figure 4:**
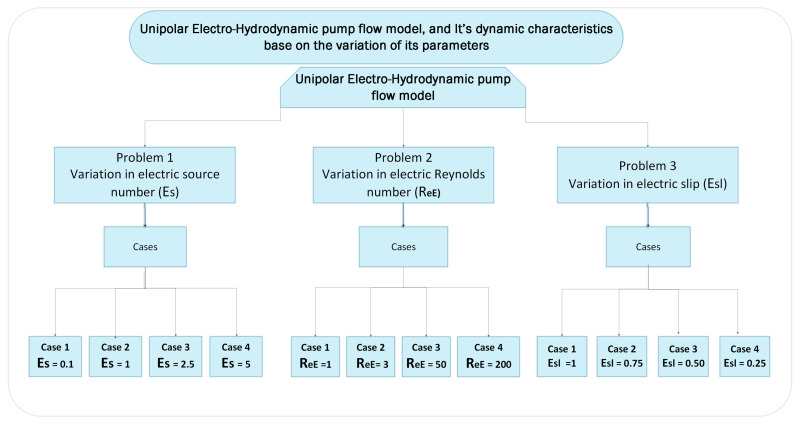
Dynamic structure of UP-EHD based on variation of its parameters.

In this section, the dynamic characteristics of unipolar hydro-electrodynamic pump flow are discussed. The discussion is split into three different problems. The problems depend on the variation of parameters of UP-EHD, i.e., electrical Reynolds number ($R_{e_{E}}$), electrical source number ($E_{s}$) and electrical slip ($E_{sl}$). The empirical results are based on 100 different independent runs. The variation in parameters is described in Figure 4.

By putting values of parameters in the model given in Equations (5)–(7) are updated for case 1 of problem 1, case 2 of problem 2 and case 3 of problem 3 as:

(29) $$\left(1+0.1E\right)\rho^{\prime }+\frac{\rho }{200}+0.1\rho^{2}=0,\;E^{\prime }-\rho =0,\;E+\phi^{\prime }=0,$$

(30) $$\left(1+E\right)\rho^{\prime }+\frac{\rho }{3}+\rho^{2}=0,\;E^{\prime }-\rho =0,\;E+\phi^{\prime }=0,$$

(31) $$\left(1+2.5E\right)\rho^{\prime }+\frac{\rho }{50}+1.25\rho^{2}=0,\;E^{\prime }-0.5\rho =0,\;E+\phi^{\prime }=0.$$

### 4.1. Problem 1: Base on Variation of Electric Slip $E_{sl}$

In this problem, the parameters electric Reynolds number ($R_{e_{E}}$) and electric source number ($E_{s}$) are kept fixed by taking their values 200 and 1, respectively. And electric slip number ($E_{sl}$) varies as $E_{sl}$ = 0.1, 1, 2.5 and 5. These values split the problem into four cases. The fitness function as described in Equations (16)–(19) for all the cases, by taking η∈ [0, 1] with step size h=0.1 with N=10, can be written as:

(32) $$e_{c1}=\left(\begin{array}{c} \frac{1}{10}\sum_{m=1}^{10}{\left(\left(1+0.1{\hat{E}}_{m}\right)\frac{d{\hat{\rho }}_{m}}{d\eta }+\frac{{\hat{\rho }}_{m}}{200}+0.1{\hat{\rho }}_{m}^{2}\right)}^{2}\\ +\frac{1}{10}\sum_{m=1}^{10}{\left(\frac{d{\hat{E}}_{m}}{d\eta }-{\hat{\rho }}_{m}\right)}^{2}+\frac{1}{10}\sum_{m=1}^{10}{\left({\hat{E}}_{m}+\frac{d{\hat{\phi }}_{m}}{d\eta }\right)}^{2}\\ +\frac{1}{3}\left({\left({\hat{\rho }}_{10}-1\right)}^{2}+{\hat{E}}_{0}^{2}+{\left({\hat{\phi }}_{10}-1\right)}^{2}\right) \end{array}\right),$$

(33) $$e_{c2}=\left(\begin{array}{c} \frac{1}{10}\sum_{m=1}^{10}{\left(\left(1+{\hat{E}}_{m}\right)\frac{d{\hat{\rho }}_{m}}{d\eta }+\frac{{\hat{\rho }}_{m}}{200}+{\hat{\rho }}_{m}^{2}\right)}^{2}\\ +\frac{1}{10}\sum_{m=1}^{10}{\left(\frac{d{\hat{E}}_{m}}{d\eta }-{\hat{\rho }}_{m}\right)}^{2}+\frac{1}{10}\sum_{m=1}^{10}{\left({\hat{E}}_{m}+\frac{d{\hat{\phi }}_{m}}{d\eta }\right)}^{2}\\ +\frac{1}{3}\left({\left({\hat{\rho }}_{10}-1\right)}^{2}+{\hat{E}}_{0}^{2}+{\left({\hat{\phi }}_{10}-1\right)}^{2}\right) \end{array}\right),$$

(34) $$e_{c3}=\left(\begin{array}{c} \frac{1}{10}\sum_{m=1}^{10}{\left(\left(1+2.5{\hat{E}}_{m}\right)\frac{d{\hat{\rho }}_{m}}{d\eta }+\frac{{\hat{\rho }}_{m}}{200}+2.5{\hat{\rho }}_{m}^{2}\right)}^{2}\\ +\frac{1}{10}\sum_{m=1}^{10}{\left(\frac{d{\hat{E}}_{m}}{d\eta }-{\hat{\rho }}_{m}\right)}^{2}+\frac{1}{10}\sum_{m=1}^{10}{\left({\hat{E}}_{m}+\frac{d{\hat{\phi }}_{m}}{d\eta }\right)}^{2}\\ +\frac{1}{3}\left({\left({\hat{\rho }}_{10}-1\right)}^{2}+{\hat{E}}_{0}^{2}+{\left({\hat{\phi }}_{10}-1\right)}^{2}\right) \end{array}\right),$$

(35) $$e_{c4}=\left(\begin{array}{c} \frac{1}{10}\sum_{m=1}^{10}{\left(\left(1+5{\hat{E}}_{m}\right)\frac{d{\hat{\rho }}_{m}}{d\eta }+\frac{{\hat{\rho }}_{m}}{200}+5{\hat{\rho }}_{m}^{2}\right)}^{2}\\ +\frac{1}{10}\sum_{m=1}^{10}{\left(\frac{d{\hat{E}}_{m}}{d\eta }-{\hat{\rho }}_{m}\right)}^{2}+\frac{1}{10}\sum_{m=1}^{10}{\left({\hat{E}}_{m}+\frac{d{\hat{\phi }}_{m}}{d\eta }\right)}^{2}\\ +\frac{1}{3}\left({\left({\hat{\rho }}_{10}-1\right)}^{2}+{\hat{E}}_{0}^{2}+{\left({\hat{\phi }}_{10}-1\right)}^{2}\right) \end{array}\right).$$

**Figure 5:**
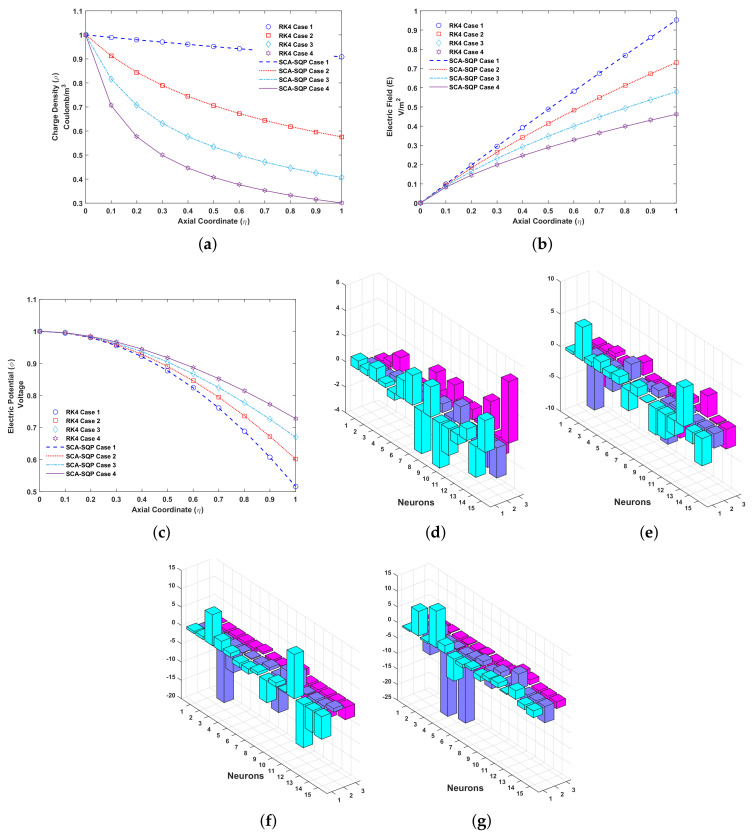
Problem 1: (**a**). Charge density (ρ)-Axial coordinate (η) graph for all cases, (**b**). Electric field (E)-Axial coordinate (η) graph for all cases, (**c**). Electric potential (ϕ)-Axial coordinate (η) graph for all cases, (**d**). Weights of case 1, (**e**). Weights of case 2, (**f**). Weights of case 3, (**g**). Weights of case 4.

The solutions for all cases of problem 1 are compared graphically with the numerical solution of the RK4 technique. RK4 is implemented through the built-in MATLAB function ode45. The comparison of the solution is shown in Figure 5. The variables of artificial neural network, known as weights, are also plotted using a 3D bar graph. The overlapping of results with RK4 shows the convergence and reliability of proposed mechanism, i.e., ANN-SCA-SQP algorithm. Figure 5a–c show the solution for ρ, E and ϕ respectively. While Figur Figure 5d–g show the weights of ANN drawn in a 3D bar graph. The detailed solutions of all the cases are given in Appendix A. Minimum (MIN), maximum (MAX), mean and standard deviation (STD) of absolute errors are given in Table 1, small variation is observed in tabulated values.

From Figure 5a, with $R_{e_{E}}=200$ and $E_{s}=1$, its observe that the value of charge density is decrease monotonically for higher values of $E_{sl}$. The charged density is maximized at collector electrode for $E_{sl}=0.1$. And if value of $E_{sl}$ is further reduce to zero it will be maximized through out the axial axis. Thus, for higher values of $R_{e_{E}}$ the maximum charge density is maintained in the entire pump.

In Figure 5b, the initial boundary condition at emitter is zero. The electric field, E, ascend from emitter, η=0, to collector electrode, η=1. The maximum value of E decrease from 0.95 approximately to 0.47 for $E_{sl}=5$, at collector electrode. The electric potential increases monotonically at the collector electrode.

The electric potential, ϕ, is initially set at 1 (Figure 5c). The potential decreases through the axial axis. At collector electrode the electric potential increase approximately from 0.5 to 0.75.

### 4.2. Problem 2: Based on the Variation of Reynolds Number $R_{e_{E}}$

In this problem, the parameters electric slip number ($E_{sl}$) and electric source number ($E_{s}$) are kept fixed by taking their values 1 for both. And electric Reynolds number ($R_{e_{E}}$) varies as $R_{e_{E}}$ =1, 3, 50 and 200. The values of $R_{e_{E}}$ establish four cases in this problem. The detail of these cases is given in Figure 4. The fitness function as described in Equations (16)–(19) for all the cases, by taking η∈ [0, 1] with step size h=0.1 with N=10, can be written as:

(36) $$e_{c1}=\left(\begin{array}{c} \frac{1}{10}\sum_{m=1}^{10}{\left(\left(1+\hat{E}\right)\frac{d\hat{\rho }}{d\eta }+\hat{\rho }+{\hat{\rho }}^{2}\right)}^{2}\\ +\frac{1}{10}\sum_{m=1}^{10}{\left(\frac{d\hat{E}}{d\eta }-\hat{\rho }\right)}^{2}+\frac{1}{10}\sum_{m=1}^{10}{\left(\hat{E}+\frac{d\hat{\phi }}{d\eta }\right)}^{2}\\ +\frac{1}{3}\left({\left(\hat{\rho }-1\right)}^{2}+{\hat{E}}^{2}+{\left(\hat{\phi }-1\right)}^{2}\right) \end{array}\right),$$

(37) $$e_{c2}=\left(\begin{array}{c} \frac{1}{10}\sum_{m=1}^{10}{\left(\left(1+\hat{E}\right)\frac{d{\hat{\rho }}_{m}}{d\eta }+\frac{{\hat{\rho }}_{m}}{3}+{\hat{\rho }}_{m}^{2}\right)}^{2}\\ +\frac{1}{10}\sum_{m=1}^{10}{\left(\frac{d{\hat{E}}_{m}}{d\eta }-{\hat{\rho }}_{m}\right)}^{2}+\frac{1}{10}\sum_{m=1}^{10}{\left({\hat{E}}_{m}+\frac{d{\hat{\phi }}_{m}}{d\eta }\right)}^{2}\\ +\frac{1}{3}\left({\left({\hat{\rho }}_{10}-1\right)}^{2}+{\hat{E}}_{m}^{2}+{\left({\hat{\phi }}_{10}-1\right)}^{2}\right) \end{array}\right),$$

(38) $$e_{c3}=\left(\begin{array}{c} \frac{1}{10}\sum_{m=1}^{10}{\left(\left(1+{\hat{E}}_{m}\right)\frac{d{\hat{\rho }}_{m}}{d\eta }+\frac{{\hat{\rho }}_{m}}{50}+{\hat{\rho }}_{m}^{2}\right)}^{2}\\ +\frac{1}{10}\sum_{m=1}^{10}{\left(\frac{d{\hat{E}}_{m}}{d\eta }-{\hat{\rho }}_{m}\right)}^{2}+\frac{1}{10}\sum_{m=1}^{10}{\left({\hat{E}}_{m}+\frac{d{\hat{\phi }}_{m}}{d\eta }\right)}^{2}\\ +\frac{1}{3}\left({\left({\hat{\rho }}_{10}-1\right)}^{2}+{\hat{E}}_{0}^{2}+{\left({\hat{\phi }}_{10}-1\right)}^{2}\right) \end{array}\right),$$

(39) $$e_{c4}=\left(\begin{array}{c} \frac{1}{10}\sum_{m=1}^{10}{\left(\left(1+{\hat{E}}_{m}\right)\frac{d{\hat{\rho }}_{m}}{d\eta }+\frac{{\hat{\rho }}_{m}}{200}+{\hat{\rho }}_{m}^{2}\right)}^{2}\\ +\frac{1}{10}\sum_{m=1}^{10}{\left(\frac{d{\hat{E}}_{m}}{d\eta }-{\hat{\rho }}_{m}\right)}^{2}+{\left({\hat{E}}_{m}+\frac{d{\hat{\phi }}_{m}}{d\eta }\right)}^{2}\\ +\frac{1}{3}\left({\left({\hat{\rho }}_{10}-1\right)}^{2}+{\hat{E}}_{0}^{2}+{\left({\hat{\phi }}_{10}-1\right)}^{2}\right) \end{array}\right).$$

**Figure 6:**
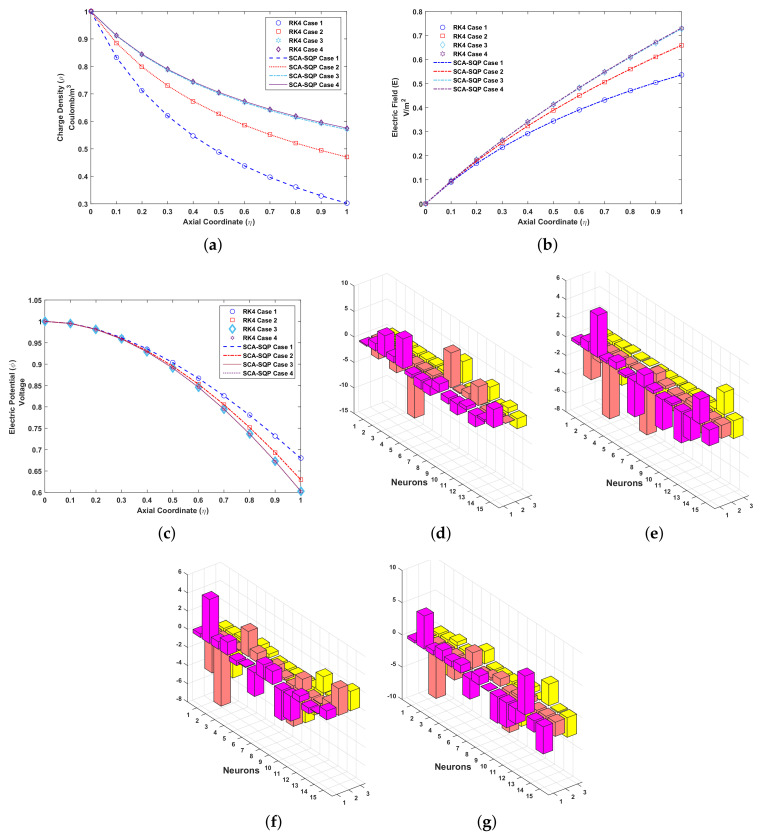
Problem 2: (**a**). Charge density (ρ)-Axial coordinate (η) graph for all cases, (**b**). Electric field (E)-Axial coordinate (η) graph for all cases, (**c**). Electric potential (ϕ)-Axial coordinate (η) graph for all cases, (**d**). Weights of case 1, (**e**). Weights of case 2, (**f**). Weights of case 3, (**g**). Weights of case 4.

The solutions for all cases of problem 2 are compared graphically with the solution of the Runge–Kutta order 4 technique. RK4 is implemented through the built-in MATLAB function ode45. The comparison of the solution is shown in Figure 6. The variables of artificial neural network, known as weights, are also plotted. The overlapping of results with RK4 shows the convergence and reliability of proposed mechanism, i.e., ANN-SCA-SQP algorithm. Figure 6a–c show the solution for ρ, E and ϕ respectively. While Figure 6d–g show the weights of ANN drawn in a 3D bar graph. The detailed solution of all the cases are given in Appendix A. Minimum (MIN), maximum (MAX), mean and standard deviation (STD) of absolute errors are given in Table 2, small variation is observed in tabulated values.

In Figure 6a, it’s observe that the charge density, ρ, decreases for all values of $R_{e_{E}}$ with axis co-ordinate. The unity is considered as initial value for charge density at emitter. For smaller values of $R_{e_{E}}$, ρ fall monotonically from unity while the difference are maximized at collector electrode, (η=1). For positive values of $R_{e_{E}}$ the inertial force dominate on electric force while for negative value of $R_{e_{E}}$ the momentum (inertial force) reduction cause the decrease in the movement of fluid.

As shown in Figure 6b, the boundary condition at the emitter is zero for electric field. A different pattern is observed for electric field by increasing $R_{e_{E}}$ from 1 to 200. At the collector, η=1, the maximum of electric field divergence is archived.

In Figure 6c, the fall of electric potential, ϕ, is observed. At the emitter the boundary condition for electric potential, ϕ, is set as 1. The value of ϕ monotonically descend from emitter to the collector electrode with increase in the value of $R_{e_{E}}$ form 1 to 200. At collector, the value of ϕ fall to 0.6.

### 4.3. Problem 3: Based on Variation of Electric Source Number $E_{s}$

In this problem, the parameters electric slip number ($E_{sl}$) and electric Reynolds number ($R_{e_{E}}$) are kept fixed by taking their values 1 for both. And electric source number ($E_{s}$) varies as $E_{s}$ = 1, 0.75, 0.5 and 0.25. These values split the problem into four cases as given in Figure 4. The simplified form of fitness function as described in Equations (16)–(19) for all the cases, by taking η∈ [0, 1] with step size h = 0.1 with N = 10, can be written as:

(40) $$e_{c1}=\left(\begin{array}{c} \frac{1}{10}\sum_{m=1}^{10}{\left(\left(1+{\hat{E}}_{m}\right)\frac{d{\hat{\rho }}_{m}}{d\eta }+\frac{{\hat{\rho }}_{m}}{50}+{\hat{\rho }}_{m}^{2}\right)}^{2}\\ +\frac{1}{10}\sum_{m=1}^{10}{\left(\frac{d{\hat{E}}_{m}}{d\eta }-{\hat{\rho }}_{m}\right)}^{2}+\frac{1}{10}\sum_{m=1}^{10}{\left({\hat{E}}_{m}+\frac{d{\hat{\phi }}_{m}}{d\eta }\right)}^{2}\\ +\frac{1}{3}\left({\left({\hat{\rho }}_{10}-1\right)}^{2}+{\hat{E}}_{0}^{2}+{\left({\hat{\phi }}_{10}-1\right)}^{2}\right) \end{array}\right),$$

(41) $$e_{c2}=\left(\begin{array}{c} \frac{1}{10}\sum_{m=1}^{10}{\left(\left(1+0.75{\hat{E}}_{m}\right)\frac{d{\hat{\rho }}_{m}}{d\eta }+\frac{{\hat{\rho }}_{m}}{50}+0.75{\hat{\rho }}_{m}^{2}\right)}^{2}\\ +\frac{1}{10}\sum_{m=1}^{10}{\left(\frac{d{\hat{E}}_{m}}{d\eta }-0.75{\hat{\rho }}_{m}\right)}^{2}+{\left({\hat{E}}_{m}+\frac{d{\hat{\phi }}_{m}}{d\eta }\right)}^{2}\\ +\frac{1}{3}\left({\left({\hat{\rho }}_{10}-1\right)}^{2}+{\hat{E}}_{0}^{2}+{\left({\hat{\phi }}_{10}-1\right)}^{2}\right) \end{array}\right),$$

(42) $$e_{c3}=\left(\begin{array}{c} i\frac{1}{10}\sum_{m=1}^{10}{\left(\left(1+0.5{\hat{E}}_{m}\right)\frac{d{\hat{\rho }}_{m}}{d\eta }+\frac{{\hat{\rho }}_{m}}{50}+0.5{\hat{\rho }}_{m}^{2}\right)}^{2}\\ +\frac{1}{10}\sum_{m=1}^{10}{\left(\frac{d{\hat{E}}_{m}}{d\eta }-0.5{\hat{\rho }}_{m}\right)}^{2}+\frac{1}{10}\sum_{m=1}^{10}{\left({\hat{E}}_{m}+\frac{d{\hat{\phi }}_{m}}{d\eta }\right)}^{2}\\ +\frac{1}{3}\left({\left({\hat{\rho }}_{10}-1\right)}^{2}+{\hat{E}}_{0}^{2}+{\left({\hat{\phi }}_{10}-1\right)}^{2}\right) \end{array}\right),$$

(43) $$e_{c4}=\left(\begin{array}{c} \frac{1}{10}\sum_{m=1}^{10}{\left(\left(1+0.25{\hat{E}}_{m}\right)\frac{d{\hat{\rho }}_{m}}{d\eta }+\frac{{\hat{\rho }}_{m}}{50}+0.25{\hat{\rho }}_{m}^{2}\right)}^{2}\\ +\frac{1}{10}\sum_{m=1}^{10}{\left(\frac{d{\hat{E}}_{m}}{d\eta }-0.25{\hat{\rho }}_{m}\right)}^{2}+\frac{1}{10}\sum_{m=1}^{10}{\left({\hat{E}}_{m}+\frac{d{\hat{\phi }}_{m}}{d\eta }\right)}^{2}\\ +\frac{1}{3}\left({\left({\hat{\rho }}_{10}-1\right)}^{2}+{\hat{E}}_{0}^{2}+{\left({\hat{\phi }}_{10}-1\right)}^{2}\right) \end{array}\right).$$

**Figure 7:**
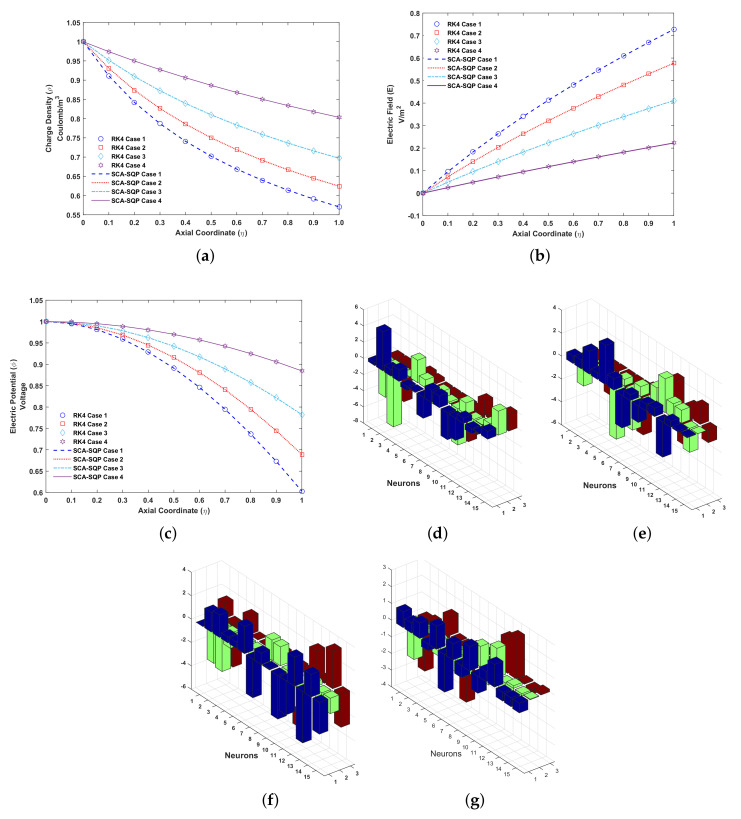
Problem 3: (**a**). Charge density (ρ)-Axial coordinate (η) graph for all cases, (**b**). Electric field (E)-Axial coordinate (η) graph for all cases, (**c**). Electric potential (ϕ)-Axial coordinate (η) graph for all cases, (**d**). Weights of case 1, (**e**). Weights of case 2, (**f**). Weights of case 3, (**g**). Weights of case 4.

The solutions for all cases of problem 3 are compared graphically with the solution of the Runge–Kutta order 4 technique. RK4 is implemented through the built-in MATLAB function ode45. The comparison of the solution is shown in Figure 7. The variables of artificial neural network, known as weights, are also plotted. The overlapping of results with RK4 shows the convergence and reliability of proposed mechanism, i.e., ANN-SCA-SQP algorithm. Figure 7a–c show the solution for ρ, E and ϕ respectively. While Figure 7d–g show the weights of ANN drawn in a 3D bar graph. The detailed solutions of all the cases are given in Appendix A. Minimum (MIN), maximum (MAX), mean and standard deviation (STD) of absolute errors are given in Table 3, small variation is observed in tabulated values. From Figure 7a its observe that the distribution of $E_{s}$ highly effect the charge density. The charge density inversely varies with $E_{s}$. With decrease in $E_{s}$, the charge density increase the collector (η=1). The profile of charge density and electric field act oppositely with variation in $E_{s}$ (Figure 7b). The electric field varies directly as $E_{s}$.

From Figure 7c, boundary condition for electric potential at emitter (η=0) is 1. At collector electrode electric potential has same behavior as charge density. But at axial axis, the variation in electric potential is different from charge density.

### 4.4. Complexity Analysis

In any technique, which generates random solutions in a given span, tuning of parameters like number of variables(neurons) and population size is a key step for performance of that technique. In such away, the proposed technique ANN-SCA-SQP generates random solutions. Therefore, performance of ANN-SCA-SQP algorithm is evaluated by variation of number of neurons and population size. In all the experiments, executed by ANN-SCA-SQP algorithm, the number of neurons are taken as 45 and population size is set 30. This combination is a vital combination. Many other are also tested but their results are not impressive. Table 4 and Table 5 reported the absolute errors for different parameters of ANN-SCA-SQP algorithm.

Table 4 reports absolute errors based on variation of number of neurons. The absolute errors for 9 neurons is between $10^{-1}$ to $10^{-3}$, which are worst errors. Absolute errors for 27 neurons lies in between $10^{-4}$ to $10^{-6}$. Absolute errors for 45 neurons are between $10^{-7}$ to $10^{-10}$ and absolute errors for 90 neurons is between $10^{-5}$ to $10^{-7}$. Overall, its seems that performance of ANN-SCA-SQP algorithm is better on 45 neurons from rest of values of neurons.

Absolute errors based of population size are reported in Table 5. The experiments are executed for 20, 30 and 40 population size. From the table its observe that the performance of ANN-SCA-SQP algorithm is much better on 30 population from the rest of population.

### 4.5. Evaluation

**Figure 8:**
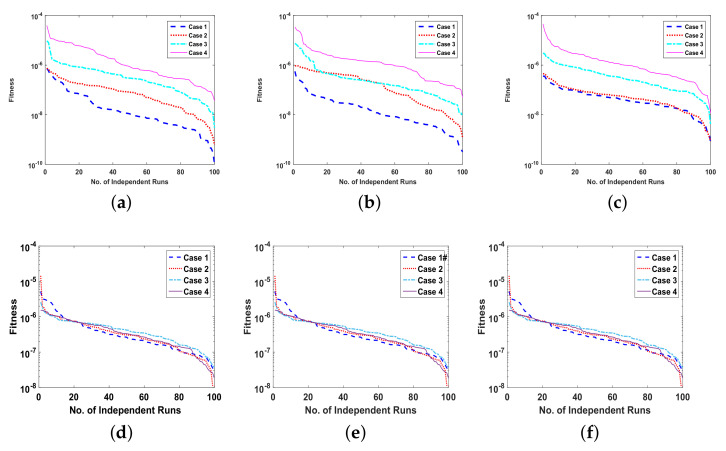

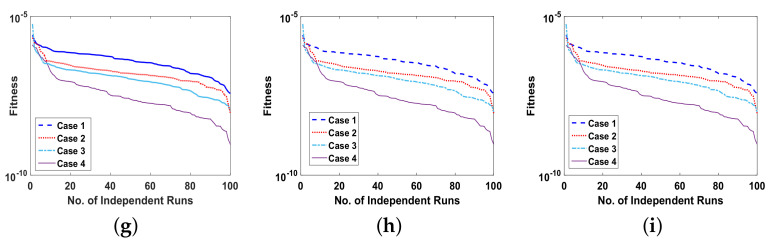
(**a**). Fitness of problem 1 all cases for ρ, (**b**). Fitness of problem 1 all cases for E, (**c**). Fitness of problem 1 all cases for ϕ, (**d**). Fitness of problem 2 all cases for ρ, (**e**). Fitness of problem 2 all cases for E, (**f**). Fitness of problem 2 all cases for ϕ, (**g**). Fitness of problem 3 all cases for ρ, (**h**). Fitness of problem 3 all cases for E, (**i**). Fitness of problem 3 all cases for ϕ.

**Figure 9:**
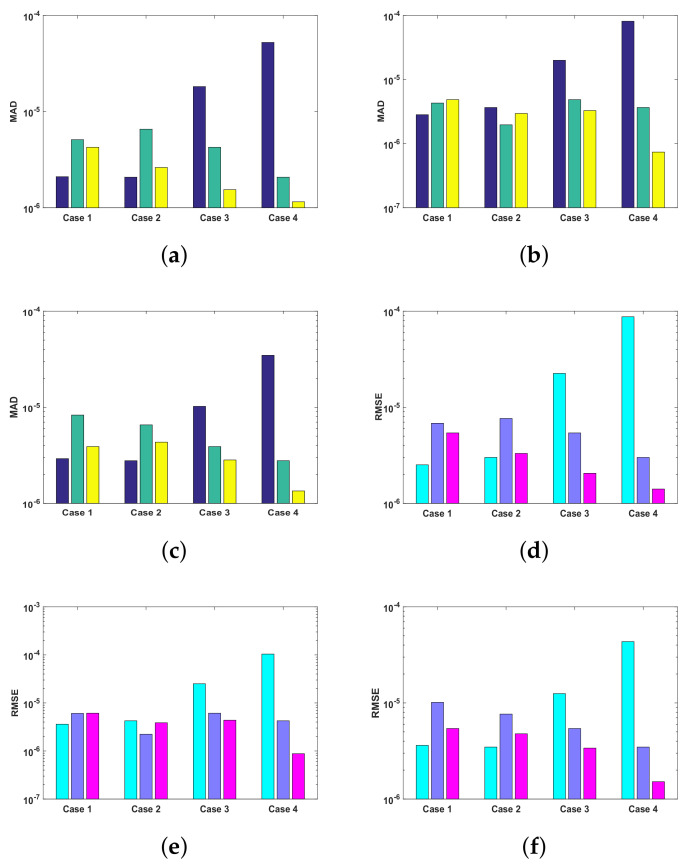

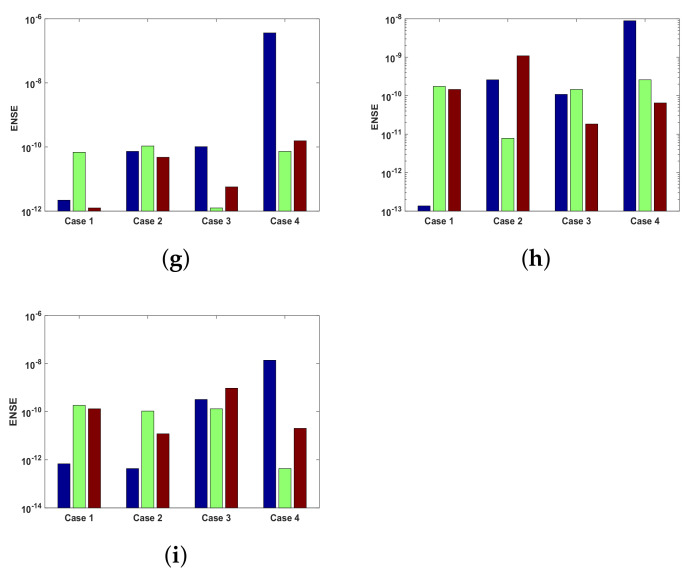
(**a**). MAD of problem 1, all cases, (**b**). MAD of problem 2, all cases, (**c**). MAD of problem 3, all cases, (**d**). RMSE of problem 1, all cases, (**e**). RMSE of problem 2, all cases, (**f**). RMSE of problem 3, all cases, (**g**). ENSE of problem 1, all cases, (**h**). ENSE of problem 2, all cases, (**i**). ENSE of problem 3, all cases.

The fitness function of all the cases of each problem is optimized by executing ANN-SCA-SQP algorithm, for the solution of UP-EHD pump flow model, for 100 independent runs. The global optimizer sine-cosine algorithm (SCA) executes in terms of ANNs to generate global fitness variables, i.e., weights, and then the weights are assigned to Sequential Quadratic Programming (SQP) as an initial point for refinement of solutions. The convergence rate of SCA gets slow down after some iterations, and the hybridization procedure of SCA and SQP increase the convergence speed. The achieved fitness of the ANN-SCA-SQP algorithm is drawn in the subfigures of Figure 8. The calculated numerical approximate solutions of ρ(η), E(η) and ϕ(η), η∈[0,1], by proposed scheme are compared with the reference solutions of Runge–Kutta order 4. The Runge–Kutta order 4 technique procedure is performed in the MATLAB software package using its built-in function known as ode45. All the solutions along with the optimization variable of ANN are presented graphically in subfigures of Figure 5, Figure 6 and Figure 7. The graphical representation of the approximated results of UP-EHD pump flow model of all the cases of each problem shows the effectiveness, reliability and validity of solutions as it overlapped RK4 solutions. For further accuracy and exactness of computed results, absolute difference (absolute error) is also calculated and is given in Table 1,Table 2 and Table 3 for problems 1, 2 and 3, respectively. The absolute error for all the cases lies between $10^{-4}$ and $10^{-8}$. The performance operators MAD, RMSE and ENSE for each dependent variable ρ, E and ϕ as defined in Equations (22)–(25) are also calculated for each case of all three problems. The calculated values are shown with the help of Bar graph in Figure 9. Each Bar in subfigures shows values of performance operator for ρ(η), E(η) and ϕ(η), respectively. The values of performance matrices address the achievement of desired values.

The Table 6 report absolute errors of hybridization of SQP with genetic algorithm (GA) and particle swarm optimization (PSO). From table, its observe that the absolute errors of ANN-SCA-SQP algorithm are between $10^{-7}$ to $10^{-9}$ while the errors for GA-SQP lies between $10^{-1}$ to $10^{-3}$ and absolute errors of PSO-SQP are in between $10^{-3}$ to $10^{-5}$. Which shows that the conjunction of SCA-SQP outperform from rest of hybridization.

### 4.6. Analysis Based on Multiple Runs

**Figure 10:**
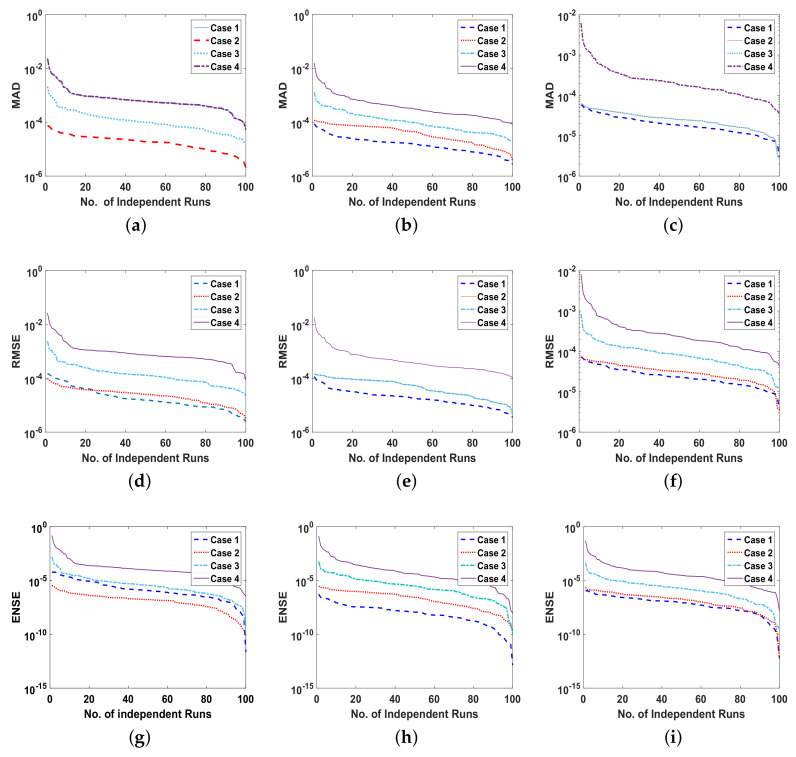
(**a**). MAD of ρ, for problem 1 all cases, (**b**). MAD of E, for problem 1 all cases, (**c**). MAD of ϕ, for problem 1 all cases, (**d**). RMSE of ρ, for problem 1 all cases, (**e**). RMSE of E, for problem 1 all cases, (**f**). RMSE of ϕ, for problem 1 all cases, (**g**). ENSE of ρ, for problem 1 all cases, (**h**). ENSE of E, for problem 1 all cases, (**i**). ENSE of ϕ, for problem 1 all cases.

**Figure 11:**
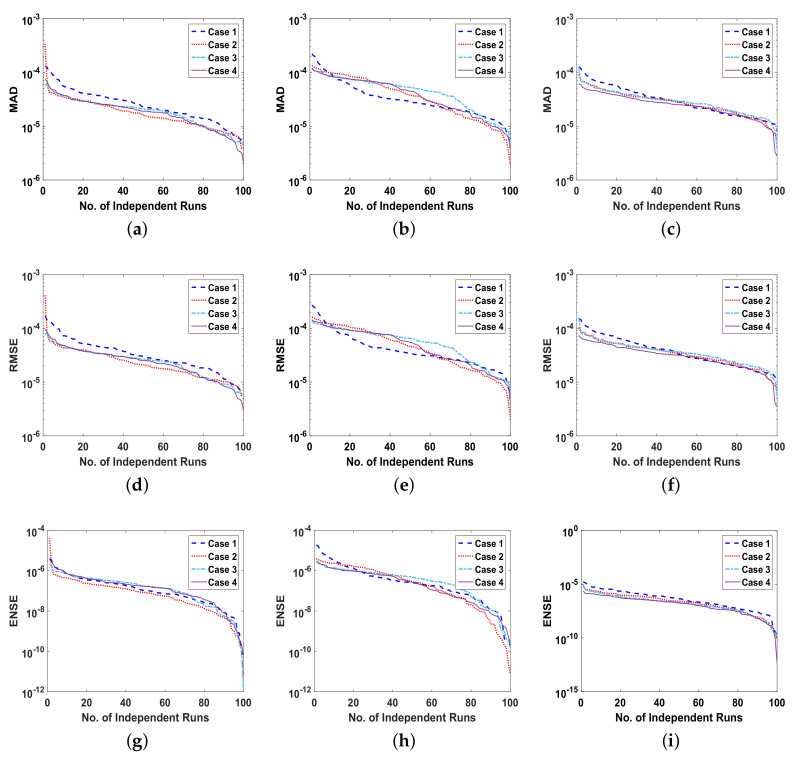
(**a**). MAD of ρ, for problem 2 all cases, (**b**). MAD of E, for problem 2 all cases, (**c**). MAD of ϕ, for problem 2all cases, (**d**). RMSE of ρ, for problem 2 all cases, (**e**). RMSE of E, for problem 2 all cases, (**f**). RMSE of ϕ, for problem 2 all cases, (**g**). ENSE of ρ, for problem 2 all cases, (**h**). ENSE of E, for problem 2 all cases, (**i**). ENSE of ϕ, for problem 2 all cases.

**Figure 12:**
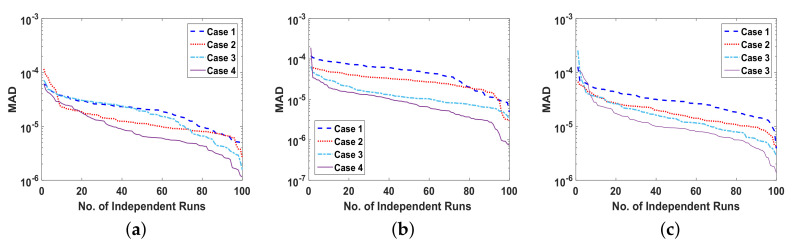

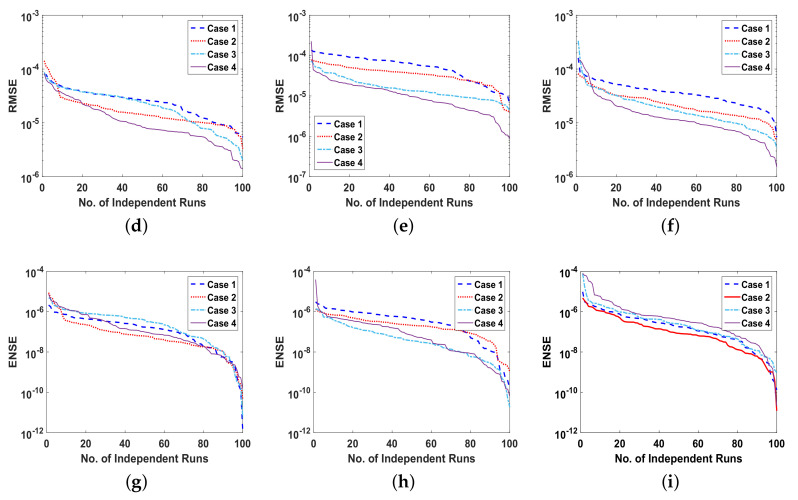
(**a**). MAD of ρ, for problem 3 all cases, (**b**). MAD of E, for problem 3 all cases, (**c**). MAD of ϕ, for problem 3, all cases, (**d**). RMSE of ρ, for problem 3 all cases, (**e**). RMSE of E, for problem 3 all cases, (**f**). RMSE of ϕ, for problem 3 all cases, (**g**). ENSE of ρ, for problem 3 all cases, (**h**). ENSE of E, for problem 3 all cases, (**i**). ENSE of ϕ, for problem 3 all cases.

The reliability of proposed ANN-SCA-SQP algorithm is based on the statistical operators. For evaluation and collection of a large set of data, the ANN-SCA-SQP algorithm is executed for 100 independent trails of UP-EHD pump model to analyze its dynamic behavior. For computed data, the statistical operators of all problems are plotted for each independent variable of the system with a semilog on the y-axis to clearly illustrate the small variation in the data. The performance matrices MAD, RMSE, and ENSE for problem 1, graphically, are given in Figure 10. Figure 11 shows MAD, RMSE, and ENSE data for problem 2 and similarly, Figure 12 shows data for problem 3. The fitness values of the scheme are also drawn in Figure 8, the fitness of problem 1 for all the cases of ρ, E, and ϕ is shown in Figure 8a–c, respectively. In same order, the Figure 8d–f are fitness of problem 2, while Figure 8g–i are fitness of problem 3. The fitness value of problem 1 lies between $10^{-4}$ to $10^{-9}$; for problem 2, the fitness values are in between $10^{-5}$ to $10^{-8}$ and for problem 3 the fitness values are in $10^{-5}$ to $10^{-10}$. The fitness values show the convergence and consistency of ANN-SCA-SQP algorithm. The reliability, effectiveness, and consistency of functionality of the ANN-SCA-SQP algorithm for the large data set, calculated for 100 runs, is evaluated in terms of statistical operators MEAN, maximum (MAX), minimum (MIN), and standard deviation (STD). The mean, MAX, MIN and STD for absolute errors is given in Table 1 for problem 1 with inputs η∈[0,1], Table 2 shows data for problem 2 and Table 3 shows data for problem 3. In tables, it’s observed that the data for all the cases are consistent. The values for MAD, RMSE, and ENSE in terms of mean and standard deviation are tabulated in Table 7. The tabulated data are distributed in three problems based on the variation of parameters of UP-EHD pump flow model, i.e., electric slip $E_{sl}$, electric source $E_{s}$, and electric Reynolds number $R_{e_{E}}$.

To inquire further correctness of the ANN-SCA-SQP algorithm the global extension of the performance matrices, named as $GMAD_{\rho }$, $GMAD_{E}$, $GMAD_{\phi }$$GRMSE_{\rho }$, $GRMSE_{E}$, $GRMSE_{\phi }$, $GENSE_{\rho }$, $GENSE_{E}$, and $GENSE_{\phi }$, were implemented as defined in Equations (26)–(28), the small variation verify the validity and correctness of the proposed methodology. The calculated results for the global version of performance matrices, in term of mean and minimum, is tabulated in Table 8. In tabulated data small variation is observed that shows consistency of ANN-SCA-SQP algorithm. The global performance matrices analysis base on their mean values and standard deviation. The variation considerably matching the desired optimal values.

**Figure 13:**
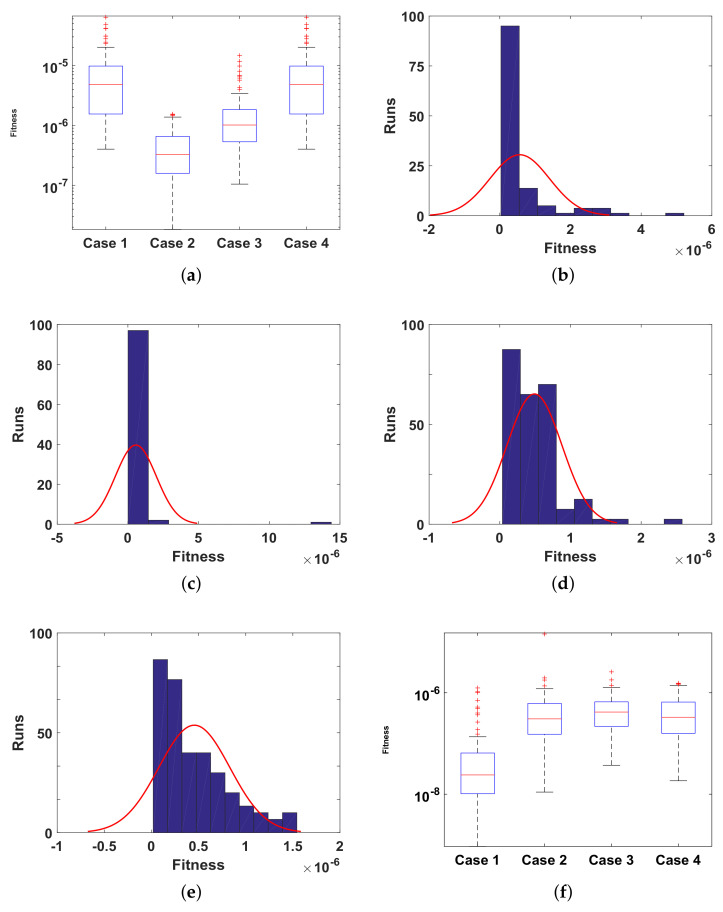
(**a**). Fitness of ρ, problem 1 all cases, (**b**). Fitness of E, problem 2 case 1, (**c**). Fitness of E, problem 2 case 2, (**d**). Fitness of E, problem 2 case 3, (**e**). Fitness of E, problem 2 case 4, (**f**). Fitness of ϕ, problem 3 all cases.

Moreover, the performance of ANN-SCA-SQP algorithm is evaluated through boxplot and histogram with normal distribution to verify its convergence. For all the cases of each problem, the boxplots and histogram with normal distribution is given in Figure 13 and its subfigures. The Figure 13a shows the fitness of charge density ρ(η) for all cases of problem 1, Figure 13b–e shows the histogram with normal distribution for fitness values of electric field E(η), case 1–4 of problem 2, respectively, and again the boxplot in Figure 13f shows the fitness values of electric potential ϕ(η) for all the cases of problem 3. The separate graph of each, with semilogarithmic scale on y-axis, is drawn in Figure 8. The Figure 8a–c shows the fitness of ρ, E and ϕ, respectively, in all the cases of problem 1. The Figure 8d–f shows the fitness of ρ, E and ϕ, respectively, in all the cases of problem 2. And The Figure 8g–j shows the fitness of ρ, E and ϕ, respectively, in all the cases of problem 3.

## 5. Conclusions

A hybrid metaheuristic solver for the solution of unipolar electro-hydrodynamic pump flow model is proposed in this work. The nonlinear UP-EHD problem arises in the field of fluid mechanics. The model of UP-EHD pump consists of first-order ordinary differential equations, solved with the global performance of the sine–cosine algorithm (SCA) and fast convergence of sequential quadratic programming (SQP) in terms of artificial neural network. The fitness/cost function of UP-EHD is formed in terms of unsupervised least square errors as the sum of squares of the residuals made in the results of every single equation of the model. The dynamic characteristics of the UP-EHD pump model are discussed in three problems, based on the variation of its parameters electric slip, Reynolds number, and electric source. The proposed scheme is comparatively studied for its validity and consistency with the Runge–Kutta order four technique. A large data set is analyzed by statistical terms means, standard deviation, minimum and maximum for absolute errors. To validate the performance of the ANN-SCA-SQP algorithm, performance matrices are implemented such as means absolute deviation, Nash–Sutcliffe efficiency and root mean square error, which verifies the precision and competency of the ANN-SCA-SQP algorithm. The ANN-SCA-SQP algorithm can also be implemented for the solution of other physical, numerical, and biological phenomena. 

## Figures and Tables

**Table 1:** Absolute errors of problem 1 for different inputs in terms of minimum, maximum, mean and standard deviation.

	Case	Mode	Absolute Errors for Inputs “η”										
			η = 0	η = 0.1	η = 0.2	η = 0.3	η = 0.4	η = 0.5	η = 0.6	η = 0.7	η = 0.8	η = 0.9	η = 1.0
ρ	1	MIN	5.30 × $10{}^{-7}$	1.58 × $10{}^{-7}$	1.41 × $10{}^{-7}$	1.40 × $10{}^{-7}$	1.54 × $10{}^{-7}$	1.71 × $10{}^{-7}$	1.36 × $10{}^{-7}$	1.20 × $10{}^{-7}$	1.28 × $10{}^{-7}$	1.51 × $10{}^{-7}$	3.57 × $10{}^{-7}$
		MAX	3.42 × $10{}^{-6}$	8.17 × $10{}^{-7}$	9.06 × $10{}^{-7}$	9.05 × $10{}^{-7}$	9.01 × $10{}^{-7}$	1.11 × $10{}^{-6}$	9.44 × $10{}^{-7}$	7.08 × $10{}^{-7}$	7.68 × $10{}^{-7}$	8.73 × $10{}^{-7}$	2.19 × $10{}^{-6}$
		MEAN	3.38 × $10{}^{-7}$	1.80 × $10{}^{-7}$	9.93 × $10{}^{-8}$	1.29 × $10{}^{-7}$	1.29 × $10{}^{-7}$	8.91 × $10{}^{-8}$	1.20 × $10{}^{-7}$	1.08 × $10{}^{-7}$	8.06 × $10{}^{-8}$	1.46 × $10{}^{-7}$	2.38 × $10{}^{-7}$
		STD	5.30 × $10{}^{-7}$	1.58 × $10{}^{-7}$	1.41 × $10{}^{-7}$	1.40 × $10{}^{-7}$	1.54 × $10{}^{-7}$	1.71 × $10{}^{-7}$	1.36 × $10{}^{-7}$	1.20 × $10{}^{-7}$	1.28 × $10{}^{-7}$	1.51 × $10{}^{-7}$	3.57 × $10{}^{-7}$
	2	MIN	4.95 × $10{}^{-9}$	3.29 × $10{}^{-8}$	4.82 × $10{}^{-9}$	7.00 × $10{}^{-9}$	9.74 × $10{}^{-9}$	5.55 × $10{}^{-9}$	6.61 × $10{}^{-10}$	5.95 × $10{}^{-9}$	7.25 × $10{}^{-9}$	1.11 × $10{}^{-8}$	1.51 × $10{}^{-8}$
		MAX	2.13 × $10{}^{-6}$	5.76 × $10{}^{-6}$	1.10 × $10{}^{-6}$	2.21 × $10{}^{-6}$	1.77 × $10{}^{-6}$	1.33 × $10{}^{-6}$	7.35 × $10{}^{-7}$	1.55 × $10{}^{-6}$	1.96 × $10{}^{-6}$	1.08 × $10{}^{-6}$	2.46 × $10{}^{-6}$
		MEAN	3.38 × $10{}^{-7}$	1.31 × $10{}^{-6}$	2.58 × $10{}^{-7}$	3.95 × $10{}^{-7}$	4.72 × $10{}^{-7}$	2.98 × $10{}^{-7}$	1.56 × $10{}^{-7}$	3.14 × $10{}^{-7}$	5.28 × $10{}^{-7}$	2.49 × $10{}^{-7}$	6.89 × $10{}^{-7}$
		STD	3.78 × $10{}^{-7}$	1.16 × $10{}^{-6}$	2.57 × $10{}^{-7}$	3.36 × $10{}^{-7}$	4.53 × $10{}^{-7}$	2.83 × $10{}^{-7}$	1.24 × $10{}^{-7}$	3.09 × $10{}^{-7}$	4.52 × $10{}^{-7}$	2.08 × $10{}^{-7}$	5.93 × $10{}^{-7}$
	3	MIN	1.76 × $10{}^{-9}$	4.97 × $10{}^{-8}$	1.10 × $10{}^{-8}$	1.61 × $10{}^{-7}$	1.74 × $10{}^{-8}$	4.27 × $10{}^{-8}$	5.28 × $10{}^{-8}$	2.12 × $10{}^{-9}$	9.66 × $10{}^{-9}$	1.68 × $10{}^{-8}$	3.05 × $10{}^{-9}$
		MAX	6.52 × $10{}^{-6}$	2.78 × $10{}^{-5}$	4.27 × $10{}^{-5}$	1.34 × $10{}^{-5}$	2.25 × $10{}^{-5}$	9.32 × $10{}^{-6}$	7.61 × $10{}^{-6}$	1.51 × $10{}^{-5}$	1.41 × $10{}^{-5}$	4.43 × $10{}^{-6}$	3.00 × $10{}^{-5}$
		MEAN	8.65 × $10{}^{-7}$	4.04 × $10{}^{-6}$	2.45 × $10{}^{-6}$	2.08 × $10{}^{-6}$	1.50 × $10{}^{-6}$	1.08 × $10{}^{-6}$	1.09 × $10{}^{-6}$	1.02 × $10{}^{-6}$	1.56 × $10{}^{-6}$	9.93 × $10{}^{-7}$	2.22 × $10{}^{-6}$
		STD	1.48 × $10{}^{-6}$	6.12 × $10{}^{-6}$	5.34 × $10{}^{-6}$	2.47 × $10{}^{-6}$	2.99 × $10{}^{-6}$	1.30 × $10{}^{-6}$	1.34 × $10{}^{-6}$	2.36 × $10{}^{-6}$	2.10 × $10{}^{-6}$	8.63 × $10{}^{-7}$	3.99 × $10{}^{-6}$
	4	MIN	1.39 × $10{}^{-9}$	5.35 × $10{}^{-9}$	1.48 × $10{}^{-7}$	1.17 × $10{}^{-7}$	4.31 × $10{}^{-8}$	2.11 × $10{}^{-8}$	8.81 × $10{}^{-8}$	4.67 × $10{}^{-8}$	1.24 × $10{}^{-7}$	1.63 × $10{}^{-7}$	2.44 × $10{}^{-7}$
		MAX	1.57 × $10{}^{-4}$	2.20 × $10{}^{-5}$	9.88 × $10{}^{-5}$	1.12 × $10{}^{-4}$	4.53 × $10{}^{-5}$	5.21 × $10{}^{-5}$	3.61 × $10{}^{-5}$	4.65 × $10{}^{-5}$	5.71 × $10{}^{-5}$	1.94 × $10{}^{-5}$	6.67 × $10{}^{-5}$
		MEAN	5.58 × $10{}^{-6}$	1.56 × $10{}^{-5}$	1.34 × $10{}^{-5}$	1.36 × $10{}^{-5}$	4.55 × $10{}^{-6}$	7.26 × $10{}^{-6}$	4.74 × $10{}^{-6}$	4.35 × $10{}^{-6}$	7.49 × $10{}^{-6}$	3.23 × $10{}^{-6}$	1.05 × $10{}^{-5}$
		STD	1.96 × $10{}^{-5}$	2.98 × $10{}^{-5}$	1.81 × $10{}^{-5}$	1.82 × $10{}^{-5}$	5.74 × $10{}^{-6}$	9.32 × $10{}^{-6}$	7.07 × $10{}^{-6}$	7.82 × $10{}^{-6}$	8.91 × $10{}^{-6}$	3.57 × $10{}^{-6}$	1.40 × $10{}^{-5}$
E	1	MIN	1.59 × $10{}^{-8}$	3.54 × $10{}^{-9}$	4.85 × $10{}^{-9}$	6.65 × $10{}^{-9}$	2.90 × $10{}^{-9}$	6.18 × $10{}^{-10}$	1.98 × $10{}^{-9}$	2.49 × $10{}^{-9}$	1.36 × $10{}^{-9}$	1.03 × $10{}^{-9}$	3.89 × $10{}^{-9}$
		MAX	3.42 × $10{}^{-6}$	8.17 × $10{}^{-7}$	9.06 × $10{}^{-7}$	9.05 × $10{}^{-7}$	9.01 × $10{}^{-7}$	1.11 × $10{}^{-6}$	9.44 × $10{}^{-7}$	7.08 × $10{}^{-7}$	7.68 × $10{}^{-7}$	8.73 × $10{}^{-7}$	2.19 × $10{}^{-6}$
		MEAN	3.38 × $10{}^{-7}$	1.80 × $10{}^{-7}$	9.93 × $10{}^{-8}$	1.29 × $10{}^{-7}$	1.29 × $10{}^{-7}$	8.91 × $10{}^{-8}$	1.20 × $10{}^{-7}$	1.08 × $10{}^{-7}$	8.06 × $10{}^{-8}$	1.46 × $10{}^{-7}$	2.38 × $10{}^{-7}$
		STD	5.30 × $10{}^{-7}$	1.58 × $10{}^{-7}$	1.41 × $10{}^{-7}$	1.40 × $10{}^{-7}$	1.54 × $10{}^{-7}$	1.71 × $10{}^{-7}$	1.36 × $10{}^{-7}$	1.20 × $10{}^{-7}$	1.28 × $10{}^{-7}$	1.51 × $10{}^{-7}$	3.57 × $10{}^{-7}$
	2	MIN	4.95 × $10{}^{-9}$	3.29 × $10{}^{-8}$	4.82 × $10{}^{-9}$	7.00 × $10{}^{-9}$	9.74 × $10{}^{-9}$	5.55 × $10{}^{-9}$	6.61 × $10{}^{-10}$	5.95 × $10{}^{-9}$	7.25 × $10{}^{-9}$	1.11 × $10{}^{-8}$	1.51 × $10{}^{-8}$
		MAX	2.13 × $10{}^{-6}$	5.76 × $10{}^{-6}$	1.10 × $10{}^{-6}$	2.21 × $10{}^{-6}$	1.77 × $10{}^{-6}$	1.33 × $10{}^{-6}$	7.35 × $10{}^{-7}$	1.55 × $10{}^{-6}$	1.96 × $10{}^{-6}$	1.08 × $10{}^{-6}$	2.46 × $10{}^{-6}$
		MEAN	3.38 × $10{}^{-7}$	1.31 × $10{}^{-6}$	2.58 × $10{}^{-7}$	3.95 × $10{}^{-7}$	4.72 × $10{}^{-7}$	2.98 × $10{}^{-7}$	1.56 × $10{}^{-7}$	3.14 × $10{}^{-7}$	5.28 × $10{}^{-7}$	2.49 × $10{}^{-7}$	6.89 × $10{}^{-7}$
		STD	3.78 × $10{}^{-7}$	1.16 × $10{}^{-6}$	2.57 × $10{}^{-7}$	3.36 × $10{}^{-7}$	4.53 × $10{}^{-7}$	2.83 × $10{}^{-7}$	1.24 × $10{}^{-7}$	3.09 × $10{}^{-7}$	4.52 × $10{}^{-7}$	2.08 × $10{}^{-7}$	5.93 × $10{}^{-7}$
	3	MIN	1.76 × $10{}^{-9}$	4.97 × $10{}^{-8}$	1.10 × $10{}^{-8}$	1.61 × $10{}^{-7}$	1.74 × $10{}^{-8}$	4.27 × $10{}^{-8}$	5.28 × $10{}^{-8}$	2.12 × $10{}^{-9}$	9.66 × $10{}^{-9}$	1.68 × $10{}^{-8}$	3.05 × $10{}^{-9}$
		MAX	6.52 × $10{}^{-6}$	2.78 × $10{}^{-5}$	4.27 × $10{}^{-5}$	1.34 × $10{}^{-5}$	2.25 × $10{}^{-5}$	9.32 × $10{}^{-6}$	7.61 × $10{}^{-6}$	1.51 × $10{}^{-5}$	1.41 × $10{}^{-5}$	4.43 × $10{}^{-6}$	3.00 × $10{}^{-5}$
		MEAN	8.65 × $10{}^{-7}$	4.04 × $10{}^{-6}$	2.45 × $10{}^{-6}$	2.08 × $10{}^{-6}$	1.50 × $10{}^{-6}$	1.08 × $10{}^{-6}$	1.09 × $10{}^{-6}$	1.02 × $10{}^{-6}$	1.56 × $10{}^{-6}$	9.93 × $10{}^{-7}$	2.22 × $10{}^{-6}$
		STD	1.48 × $10{}^{-6}$	6.12 × $10{}^{-6}$	5.34 × $10{}^{-6}$	2.47 × $10{}^{-6}$	2.99 × $10{}^{-6}$	1.30 × $10{}^{-6}$	1.34 × $10{}^{-6}$	2.36 × $10{}^{-6}$	2.10 × $10{}^{-6}$	8.63 × $10{}^{-7}$	3.99 × $10{}^{-6}$
	4	MIN	1.39 × $10{}^{-9}$	5.35 × $10{}^{-9}$	1.48 × $10{}^{-7}$	1.17 × $10{}^{-7}$	4.31 × $10{}^{-8}$	2.11 × $10{}^{-8}$	8.81 × $10{}^{-8}$	4.67 × $10{}^{-8}$	1.24 × $10{}^{-7}$	1.63 × $10{}^{-7}$	2.44 × $10{}^{-7}$
		MAX	1.57 × $10{}^{-4}$	2.20 × $10{}^{-4}$	9.88 × $10{}^{-5}$	1.12 × $10{}^{-4}$	4.53 × $10{}^{-5}$	5.21 × $10{}^{-5}$	3.61 × $10{}^{-5}$	4.65 × $10{}^{-5}$	5.71 × $10{}^{-5}$	1.94 × $10{}^{-5}$	6.67 × $10{}^{-5}$
		MEAN	5.58 × $10{}^{-6}$	1.56 × $10{}^{-5}$	1.34 × $10{}^{-5}$	1.36 × $10{}^{-5}$	4.55 × $10{}^{-6}$	7.26 × $10{}^{-6}$	4.74 × $10{}^{-6}$	4.35 × $10{}^{-6}$	7.49 × $10{}^{-6}$	3.23 × $10{}^{-6}$	1.05 × $10{}^{-5}$
		STD	1.96 × $10{}^{-5}$	2.98 × $10{}^{-5}$	1.81 × $10{}^{-5}$	1.82 × $10{}^{-5}$	5.74 × $10{}^{-6}$	9.32 × $10{}^{-6}$	7.07 × $10{}^{-6}$	7.82 × $10{}^{-6}$	8.91 × $10{}^{-6}$	3.57 × $10{}^{-6}$	1.40 × $10{}^{-5}$
ϕ	1	MIN	1.59 × $10{}^{-8}$	3.54 × $10{}^{-9}$	4.85 × $10{}^{-9}$	6.65 × $10{}^{-9}$	2.90 × $10{}^{-9}$	6.18 × $10{}^{-10}$	1.98 × $10{}^{-9}$	2.49 × $10{}^{-9}$	1.36 × $10{}^{-9}$	1.03 × $10{}^{-9}$	3.89 × $10{}^{-9}$
		MAX	3.42 × $10{}^{-6}$	8.17 × $10{}^{-7}$	9.06 × $10{}^{-7}$	9.05 × $10{}^{-7}$	9.01 × $10{}^{-7}$	1.11 × $10{}^{-6}$	9.44 × $10{}^{-7}$	7.08 × $10{}^{-7}$	7.68 × $10{}^{-7}$	8.73 × $10{}^{-7}$	2.19 × $10{}^{-6}$
		MEAN	3.38 × $10{}^{-7}$	1.80 × $10{}^{-7}$	9.93 × $10{}^{-8}$	1.29 × $10{}^{-7}$	1.29 × $10{}^{-7}$	8.91 × $10{}^{-8}$	1.20 × $10{}^{-7}$	1.08 × $10{}^{-7}$	8.06 × $10{}^{-8}$	1.46 × $10{}^{-7}$	2.38 × $10{}^{-7}$
		STD	5.30 × $10{}^{-7}$	1.58 × $10{}^{-7}$	1.41 × $10{}^{-7}$	1.40 × $10{}^{-7}$	1.54 × $10{}^{-7}$	1.71 × $10{}^{-7}$	1.36 × $10{}^{-7}$	1.20 × $10{}^{-7}$	1.28 × $10{}^{-7}$	1.51 × $10{}^{-7}$	3.57 × $10{}^{-7}$
	2	MIN	4.95 × $10{}^{-9}$	3.29 × $10{}^{-8}$	4.82 × $10{}^{-9}$	7.00 × $10{}^{-9}$	9.74 × $10{}^{-9}$	5.55 × $10{}^{-9}$	6.61 × $10{}^{-10}$	5.95 × $10{}^{-9}$	7.25 × $10{}^{-9}$	1.11 × $10{}^{-8}$	1.51 × $10{}^{-8}$
		MAX	2.13 × $10{}^{-6}$	5.76 × $10{}^{-6}$	1.10 × $10{}^{-6}$	2.21 × $10{}^{-6}$	1.77 × $10{}^{-6}$	1.33 × $10{}^{-6}$	7.35 × $10{}^{-7}$	1.55 × $10{}^{-6}$	1.96 × $10{}^{-6}$	1.08 × $10{}^{-6}$	2.46 × $10{}^{-6}$
		MEAN	3.38 × $10{}^{-7}$	1.31 × $10{}^{-6}$	2.58 × $10{}^{-7}$	3.95 × $10{}^{-7}$	4.72 × $10{}^{-7}$	2.98 × $10{}^{-7}$	1.56 × $10{}^{-7}$	3.14 × $10{}^{-7}$	5.28 × $10{}^{-7}$	2.49 × $10{}^{-7}$	6.89 × $10{}^{-7}$
		STD	3.78 × $10{}^{-7}$	1.16 × $10{}^{-6}$	2.57 × $10{}^{-7}$	3.36 × $10{}^{-7}$	4.53 × $10{}^{-7}$	2.83 × $10{}^{-7}$	1.24 × $10{}^{-7}$	3.09 × $10{}^{-7}$	4.52 × $10{}^{-7}$	2.08 × $10{}^{-7}$	5.93 × $10{}^{-7}$
	3	MIN	1.76 × $10{}^{-9}$	4.97 × $10{}^{-8}$	1.10 × $10{}^{-8}$	1.61 × $10{}^{-7}$	1.74 × $10{}^{-8}$	4.27 × $10{}^{-8}$	5.28 × $10{}^{-8}$	2.12 × $10{}^{-9}$	9.66 × $10{}^{-9}$	1.68 × $10{}^{-8}$	3.05 × $10{}^{-9}$
		MAX	6.52 × $10{}^{-6}$	2.78 × $10{}^{-5}$	4.27 × $10{}^{-5}$	1.34 × $10{}^{-5}$	2.25 × $10{}^{-5}$	9.32 × $10{}^{-6}$	7.61 × $10{}^{-6}$	1.51 × $10{}^{-5}$	1.41 × $10{}^{-5}$	4.43 × $10{}^{-6}$	3.00 × $10{}^{-5}$
		MEAN	8.65 × $10{}^{-7}$	4.04 × $10{}^{-6}$	2.45 × $10{}^{-6}$	2.08 × $10{}^{-6}$	1.50 × $10{}^{-6}$	1.08 × $10{}^{-6}$	1.09 × $10{}^{-6}$	1.02 × $10{}^{-6}$	1.56 × $10{}^{-6}$	9.93 × $10{}^{-7}$	2.22 × $10{}^{-6}$
		STD	1.48 × $10{}^{-6}$	6.12 × $10{}^{-6}$	5.34 × $10{}^{-6}$	2.47 × $10{}^{-6}$	2.99 × $10{}^{-6}$	1.30 × $10{}^{-6}$	1.34 × $10{}^{-6}$	2.36 × $10{}^{-6}$	2.10 × $10{}^{-6}$	8.63 × $10{}^{-7}$	3.99 × $10{}^{-6}$
	4	MIN	1.39 × $10{}^{-9}$	5.35 × $10{}^{-9}$	1.48 × $10{}^{-7}$	1.17 × $10{}^{-7}$	4.31 × $10{}^{-8}$	2.11 × $10{}^{-8}$	8.81 × $10{}^{-8}$	4.67 × $10{}^{-8}$	1.24 × $10{}^{-7}$	1.63 × $10{}^{-7}$	2.44 × $10{}^{-7}$
		MAX	1.57 × $10{}^{-4}$	2.20 × $10{}^{-4}$	9.88 × $10{}^{-5}$	1.12 × $10{}^{-4}$	4.53 × $10{}^{-5}$	5.21 × $10{}^{-5}$	3.61 × $10{}^{-5}$	4.65 × $10{}^{-5}$	5.71 × $10{}^{-5}$	1.94 × $10{}^{-5}$	6.67 × $10{}^{-5}$
		MEAN	5.58 × $10{}^{-6}$	1.56 × $10{}^{-5}$	1.34 × $10{}^{-5}$	1.36 × $10{}^{-5}$	4.55 × $10{}^{-6}$	7.26 × $10{}^{-6}$	4.74 × $10{}^{-6}$	4.35 × $10{}^{-6}$	7.49 × $10{}^{-6}$	3.23 × $10{}^{-6}$	1.05 × $10{}^{-5}$
		STD	1.96 × $10{}^{-5}$	2.98 × $10{}^{-5}$	1.81 × $10{}^{-5}$	1.82 × $10{}^{-5}$	5.74 × $10{}^{-6}$	9.32 × $10{}^{-6}$	7.07 × $10{}^{-6}$	7.82 × $10{}^{-6}$	8.91 × $10{}^{-6}$	3.57 × $10{}^{-6}$	1.40 × $10{}^{-5}$

**Table 2:** Absolute errors of problem 2 for different inputs in terms of minimum, maximum, mean and standard deviation.

	Case	Mode	Absolute Errors for Inputs “η”										
			η = 0	η = 0.1	η = 0.2	η = 0.3	η = 0.4	η = 0.5	η = 0.6	η = 0.7	η = 0.8	η = 0.9	η = 1.0
ρ	1	MIN	5.920 × $10{}^{-9}$	8.090 × $10{}^{-8}$	8.020 × $10{}^{-9}$	5.010 × $10{}^{-9}$	5.680 × $10{}^{-9}$	1.040 × $10{}^{-8}$	2.340 × $10{}^{-9}$	1.620 × $10{}^{-9}$	1.430 × $10{}^{-8}$	1.860 × $10{}^{-8}$	2.630 × $10{}^{-8}$
		MAX	2.700 × $10{}^{-6}$	1.550 × $10{}^{-5}$	7.040 × $10{}^{-6}$	5.210 × $10{}^{-6}$	5.370 × $10{}^{-6}$	4.250 × $10{}^{-6}$	1.950 × $10{}^{-6}$	4.310 × $10{}^{-6}$	5.920 × $10{}^{-6}$	2.520 × $10{}^{-6}$	8.060 × $10{}^{-6}$
		MEAN	2.760 × $10{}^{-7}$	1.440 × $10{}^{-6}$	6.470 × $10{}^{-7}$	4.990 × $10{}^{-7}$	5.100 × $10{}^{-7}$	4.120 × $10{}^{-7}$	2.510 × $10{}^{-7}$	3.730 × $10{}^{-7}$	6.230 × $10{}^{-7}$	3.030 × $10{}^{-7}$	8.630 × $10{}^{-7}$
		STD	4.930 × $10{}^{-7}$	2.350 × $10{}^{-6}$	1.080 × $10{}^{-6}$	8.260 × $10{}^{-7}$	8.540 × $10{}^{-7}$	6.980 × $10{}^{-7}$	3.250 × $10{}^{-7}$	7.370 × $10{}^{-7}$	1.020 × $10{}^{-6}$	3.760 × $10{}^{-7}$	1.470 × $10{}^{-6}$
	2	MIN	5.500 × $10{}^{-10}$	1.040 × $10{}^{-8}$	8.840 × $10{}^{-9}$	7.560 × $10{}^{-9}$	4.840 × $10{}^{-9}$	4.830 × $10{}^{-9}$	9.580 × $10{}^{-9}$	1.200 × $10{}^{-9}$	9.190 × $10{}^{-9}$	1.890 × $10{}^{-9}$	1.650 × $10{}^{-8}$
		MAX	1.410 × $10{}^{-5}$	5.000 × $10{}^{-5}$	1.440 × $10{}^{-6}$	1.450 × $10{}^{-5}$	1.870 × $10{}^{-5}$	3.040 × $10{}^{-6}$	2.720 × $10{}^{-6}$	1.480 × $10{}^{-5}$	1.380 × $10{}^{-5}$	1.070 × $10{}^{-6}$	2.450 × $10{}^{-5}$
		MEAN	4.360 × $10{}^{-7}$	1.750 × $10{}^{-6}$	2.560 × $10{}^{-7}$	5.440 × $10{}^{-7}$	6.300 × $10{}^{-7}$	2.730 × $10{}^{-7}$	1.800 × $10{}^{-7}$	4.550 × $10{}^{-7}$	5.900 × $10{}^{-7}$	2.050 × $10{}^{-7}$	8.590 × $10{}^{-7}$
		STD	1.420 × $10{}^{-6}$	5.020 × $10{}^{-6}$	2.800 × $10{}^{-7}$	1.440 × $10{}^{-6}$	1.890 × $10{}^{-6}$	3.730 × $10{}^{-7}$	2.790 × $10{}^{-7}$	1.480 × $10{}^{-6}$	1.410 × $10{}^{-6}$	1.640 × $10{}^{-7}$	2.460 × $10{}^{-6}$
	3	MIN	6.710 × $10{}^{-9}$	3.990 × $10{}^{-8}$	1.180 × $10{}^{-9}$	2.350 × $10{}^{-8}$	1.360 × $10{}^{-8}$	1.040 × $10{}^{-8}$	3.080 × $10{}^{-9}$	3.450 × $10{}^{-9}$	1.860 × $10{}^{-8}$	2.720 × $10{}^{-8}$	3.100 × $10{}^{-8}$
		MAX	1.930 × $10{}^{-6}$	6.980 × $10{}^{-6}$	1.840 × $10{}^{-6}$	3.280 × $10{}^{-6}$	2.220 × $10{}^{-6}$	1.940 × $10{}^{-6}$	1.350 × $10{}^{-6}$	2.170 × $10{}^{-6}$	2.720 × $10{}^{-6}$	9.430 × $10{}^{-7}$	4.260 × $10{}^{-6}$
		MEAN	3.840 × $10{}^{-7}$	1.390 × $10{}^{-6}$	3.160 × $10{}^{-7}$	4.100 × $10{}^{-7}$	5.050 × $10{}^{-7}$	3.460 × $10{}^{-7}$	1.750 × $10{}^{-7}$	3.130 × $10{}^{-7}$	5.560 × $10{}^{-7}$	2.900 × $10{}^{-7}$	7.020 × $10{}^{-7}$
		STD	3.520 × $10{}^{-7}$	1.060 × $10{}^{-6}$	3.500 × $10{}^{-7}$	3.660 × $10{}^{-7}$	4.300 × $10{}^{-7}$	3.310 × $10{}^{-7}$	1.630 × $10{}^{-7}$	2.880 × $10{}^{-7}$	4.810 × $10{}^{-7}$	2.140 × $10{}^{-7}$	6.130 × $10{}^{-7}$
	4	MIN	4.950 × $10{}^{-9}$	3.290 × $10{}^{-8}$	4.820 × $10{}^{-9}$	7.000 × $10{}^{-9}$	9.740 × $10{}^{-9}$	5.550 × $10{}^{-9}$	6.610 × $10{}^{-10}$	5.950 × $10{}^{-9}$	7.250 × $10{}^{-9}$	1.110 × $10{}^{-8}$	1.510 × $10{}^{-8}$
		MAX	2.130 × $10{}^{-6}$	5.760 × $10{}^{-6}$	1.100 × $10{}^{-6}$	2.210 × $10{}^{-6}$	1.770 × $10{}^{-6}$	1.330 × $10{}^{-6}$	7.350 × $10{}^{-7}$	1.550 × $10{}^{-6}$	1.960 × $10{}^{-6}$	1.080 × $10{}^{-6}$	2.460 × $10{}^{-6}$
		MEAN	3.380 × $10{}^{-7}$	1.310 × $10{}^{-6}$	2.580 × $10{}^{-7}$	3.950 × $10{}^{-7}$	4.720 × $10{}^{-7}$	2.980 × $10{}^{-7}$	1.560 × $10{}^{-7}$	3.140 × $10{}^{-7}$	5.280 × $10{}^{-7}$	2.490 × $10{}^{-7}$	6.890 × $10{}^{-7}$
		STD	3.780 × $10{}^{-7}$	1.160 × $10{}^{-6}$	2.570 × $10{}^{-7}$	3.360 × $10{}^{-7}$	4.530 × $10{}^{-7}$	2.830 × $10{}^{-7}$	1.240 × $10{}^{-7}$	3.090 × $10{}^{-7}$	4.520 × $10{}^{-7}$	2.080 × $10{}^{-7}$	5.930 × $10{}^{-7}$
E	1	MIN	5.920 × $10{}^{-9}$	8.090 × $10{}^{-8}$	8.020 × $10{}^{-9}$	5.010 × $10{}^{-9}$	5.680 × $10{}^{-9}$	1.040 × $10{}^{-8}$	2.340 × $10{}^{-9}$	1.620 × $10{}^{-9}$	1.430 × $10{}^{-8}$	1.860 × $10{}^{-8}$	2.630 × $10{}^{-8}$
		MAX	2.700 × $10{}^{-6}$	1.550 × $10{}^{-5}$	7.040 × $10{}^{-6}$	5.210 × $10{}^{-6}$	5.370 × $10{}^{-6}$	4.250 × $10{}^{-6}$	1.950 × $10{}^{-6}$	4.310 × $10{}^{-6}$	5.920 × $10{}^{-6}$	2.520 × $10{}^{-6}$	8.060 × $10{}^{-6}$
		MEAN	2.760 × $10{}^{-7}$	1.440 × $10{}^{-6}$	6.470 × $10{}^{-7}$	4.990 × $10{}^{-7}$	5.100 × $10{}^{-7}$	4.120 × $10{}^{-7}$	2.510 × $10{}^{-7}$	3.730 × $10{}^{-7}$	6.230 × $10{}^{-7}$	3.030 × $10{}^{-7}$	8.630 × $10{}^{-7}$
		STD	4.930 × $10{}^{-7}$	2.350 × $10{}^{-6}$	1.080 × $10{}^{-6}$	8.260 × $10{}^{-7}$	8.540 × $10{}^{-7}$	6.980 × $10{}^{-7}$	3.250 × $10{}^{-7}$	7.370 × $10{}^{-7}$	1.020 × $10{}^{-6}$	3.760 × $10{}^{-7}$	1.470 × $10{}^{-6}$
	2	MIN	5.500 × $10{}^{-10}$	1.040 × $10{}^{-8}$	8.840 × $10{}^{-9}$	7.560 × $10{}^{-9}$	4.840 × $10{}^{-9}$	4.830 × $10{}^{-9}$	9.580 × $10{}^{-9}$	1.200 × $10{}^{-9}$	9.190 × $10{}^{-9}$	1.890 × $10{}^{-9}$	1.650 × $10{}^{-8}$
		MAX	1.410 × $10{}^{-5}$	5.000 × $10{}^{-5}$	1.440 × $10{}^{-6}$	1.450 × $10{}^{-5}$	1.870 × $10{}^{-5}$	3.040 × $10{}^{-6}$	2.720 × $10{}^{-6}$	1.480 × $10{}^{-5}$	1.380 × $10{}^{-5}$	1.070 × $10{}^{-6}$	2.450 × $10{}^{-5}$
		MEAN	4.360 × $10{}^{-7}$	1.750 × $10{}^{-6}$	2.560 × $10{}^{-7}$	5.440 × $10{}^{-7}$	6.300 × $10{}^{-7}$	2.730 × $10{}^{-7}$	1.800 × $10{}^{-7}$	4.550 × $10{}^{-7}$	5.900 × $10{}^{-7}$	2.050 × $10{}^{-7}$	8.590 × $10{}^{-7}$
		STD	1.420 × $10{}^{-6}$	5.020 × $10{}^{-6}$	2.800 × $10{}^{-7}$	1.440 × $10{}^{-6}$	1.890 × $10{}^{-6}$	3.730 × $10{}^{-7}$	2.790 × $10{}^{-7}$	1.480 × $10{}^{-6}$	1.410 × $10{}^{-6}$	1.640 × $10{}^{-7}$	2.460 × $10{}^{-6}$
	3	MIN	6.710 × $10{}^{-9}$	3.990 × $10{}^{-8}$	1.180 × $10{}^{-9}$	2.350 × $10{}^{-8}$	1.360 × $10{}^{-8}$	1.040 × $10{}^{-8}$	3.080 × $10{}^{-9}$	3.450 × $10{}^{-9}$	1.860 × $10{}^{-8}$	2.720 × $10{}^{-8}$	3.100 × $10{}^{-8}$
		MAX	1.930 × $10{}^{-6}$	6.980 × $10{}^{-6}$	1.840 × $10{}^{-6}$	3.280 × $10{}^{-6}$	2.220 × $10{}^{-6}$	1.940 × $10{}^{-6}$	1.350 × $10{}^{-6}$	2.170 × $10{}^{-6}$	2.720 × $10{}^{-6}$	9.430 × $10{}^{-7}$	4.260 × $10{}^{-6}$
		MEAN	3.840 × $10{}^{-7}$	1.390 × $10{}^{-6}$	3.160 × $10{}^{-7}$	4.100 × $10{}^{-7}$	5.050 × $10{}^{-7}$	3.460 × $10{}^{-7}$	1.750 × $10{}^{-7}$	3.130 × $10{}^{-7}$	5.560 × $10{}^{-7}$	2.900 × $10{}^{-7}$	7.020 × $10{}^{-7}$
		STD	3.520 × $10{}^{-7}$	1.060 × $10{}^{-6}$	3.500 × $10{}^{-7}$	3.660 × $10{}^{-7}$	4.300 × $10{}^{-7}$	3.310 × $10{}^{-7}$	1.630 × $10{}^{-7}$	2.880 × $10{}^{-7}$	4.810 × $10{}^{-7}$	2.140 × $10{}^{-7}$	6.130 × $10{}^{-7}$
	4	MIN	4.950 × $10{}^{-9}$	3.290 × $10{}^{-8}$	4.820 × $10{}^{-9}$	7.000 × $10{}^{-9}$	9.740 × $10{}^{-9}$	5.550 × $10{}^{-9}$	6.610 × $10{}^{-10}$	5.950 × $10{}^{-9}$	7.250 × $10{}^{-9}$	1.110 × $10{}^{-8}$	1.510 × $10{}^{-8}$
		MAX	2.130 × $10{}^{-6}$	5.760 × $10{}^{-6}$	1.100 × $10{}^{-6}$	2.210 × $10{}^{-6}$	1.770 × $10{}^{-6}$	1.330 × $10{}^{-6}$	7.350 × $10{}^{-7}$	1.550 × $10{}^{-6}$	1.960 × $10{}^{-6}$	1.080 × $10{}^{-6}$	2.460 × $10{}^{-6}$
		MEAN	3.380 × $10{}^{-7}$	1.310 × $10{}^{-6}$	2.580 × $10{}^{-7}$	3.950 × $10{}^{-7}$	4.720 × $10{}^{-7}$	2.980 × $10{}^{-7}$	1.560 × $10{}^{-7}$	3.140 × $10{}^{-7}$	5.280 × $10{}^{-7}$	2.490 × $10{}^{-7}$	6.890 × $10{}^{-7}$
		STD	3.780 × $10{}^{-7}$	1.160 × $10{}^{-6}$	2.570 × $10{}^{-7}$	3.360 × $10{}^{-7}$	4.530 × $10{}^{-7}$	2.830 × $10{}^{-7}$	1.240 × $10{}^{-7}$	3.090 × $10{}^{-7}$	4.520 × $10{}^{-7}$	2.080 × $10{}^{-7}$	5.930 × $10{}^{-7}$
ϕ	1	MIN	5.920 × $10{}^{-9}$	8.090 × $10{}^{-8}$	8.020 × $10{}^{-9}$	5.010 × $10{}^{-9}$	5.680 × $10{}^{-9}$	1.040 × $10{}^{-8}$	2.340 × $10{}^{-9}$	1.620 × $10{}^{-9}$	1.430 × $10{}^{-8}$	1.860 × $10{}^{-8}$	2.630 × $10{}^{-8}$
		MAX	2.700 × $10{}^{-6}$	1.550 × $10{}^{-5}$	7.040 × $10{}^{-6}$	5.210 × $10{}^{-6}$	5.370 × $10{}^{-6}$	4.250 × $10{}^{-6}$	1.950 × $10{}^{-6}$	4.310 × $10{}^{-6}$	5.920 × $10{}^{-6}$	2.520 × $10{}^{-6}$	8.060 × $10{}^{-6}$
		MEAN	2.760 × $10{}^{-7}$	1.440 × $10{}^{-6}$	6.470 × $10{}^{-7}$	4.990 × $10{}^{-7}$	5.100 × $10{}^{-7}$	4.120 × $10{}^{-7}$	2.510 × $10{}^{-7}$	3.730 × $10{}^{-7}$	6.230 × $10{}^{-7}$	3.030 × $10{}^{-7}$	8.630 × $10{}^{-7}$
		STD	4.930 × $10{}^{-7}$	2.350 × $10{}^{-6}$	1.080 × $10{}^{-6}$	8.260 × $10{}^{-7}$	8.540 × $10{}^{-7}$	6.980 × $10{}^{-7}$	3.250 × $10{}^{-7}$	7.370 × $10{}^{-7}$	1.020 × $10{}^{-6}$	3.760 × $10{}^{-7}$	1.470 × $10{}^{-6}$
	2	MIN	5.500 × $10{}^{-10}$	1.040 × $10{}^{-8}$	8.840 × $10{}^{-9}$	7.560 × $10{}^{-9}$	4.840 × $10{}^{-9}$	4.830 × $10{}^{-9}$	9.580 × $10{}^{-9}$	1.200 × $10{}^{-9}$	9.190 × $10{}^{-9}$	1.890 × $10{}^{-9}$	1.650 × $10{}^{-8}$
		MAX	1.410 × $10{}^{-5}$	5.000 × $10{}^{-5}$	1.440 × $10{}^{-6}$	1.450 × $10{}^{-5}$	1.870 × $10{}^{-5}$	3.040 × $10{}^{-6}$	2.720 × $10{}^{-6}$	1.480 × $10{}^{-5}$	1.380 × $10{}^{-5}$	1.070 × $10{}^{-6}$	2.450 × $10{}^{-5}$
		MEAN	4.360 × $10{}^{-7}$	1.750 × $10{}^{-6}$	2.560 × $10{}^{-7}$	5.440 × $10{}^{-7}$	6.300 × $10{}^{-7}$	2.730 × $10{}^{-7}$	1.800 × $10{}^{-7}$	4.550 × $10{}^{-7}$	5.900 × $10{}^{-7}$	2.050 × $10{}^{-7}$	8.590 × $10{}^{-7}$
		STD	1.420 × $10{}^{-6}$	5.020 × $10{}^{-6}$	2.800 × $10{}^{-7}$	1.440 × $10{}^{-6}$	1.890 × $10{}^{-6}$	3.730 × $10{}^{-7}$	2.790 × $10{}^{-7}$	1.480 × $10{}^{-6}$	1.410 × $10{}^{-6}$	1.640 × $10{}^{-7}$	2.460 × $10{}^{-6}$
	3	MIN	6.710 × $10{}^{-9}$	3.990 × $10{}^{-8}$	1.180 × $10{}^{-9}$	2.350 × $10{}^{-8}$	1.360 × $10{}^{-8}$	1.040 × $10{}^{-8}$	3.08 × $10{}^{-9}$	3.450 × $10{}^{-9}$	1.860 × $10{}^{-8}$	2.720 × $10{}^{-8}$	3.100 × $10{}^{-8}$
		MAX	1.930 × $10{}^{-6}$	6.980 × $10{}^{-6}$	1.840 × $10{}^{-6}$	3.280 × $10{}^{-6}$	2.220 × $10{}^{-6}$	1.940 × $10{}^{-6}$	1.350 × $10{}^{-6}$	2.170 × $10{}^{-6}$	2.720 × $10{}^{-6}$	9.430 × $10{}^{-7}$	4.260 × $10{}^{-6}$
		MEAN	3.840 × $10{}^{-7}$	1.390 × $10{}^{-6}$	3.160 × $10{}^{-7}$	4.100 × $10{}^{-7}$	5.050 × $10{}^{-7}$	3.460 × $10{}^{-7}$	1.750 × $10{}^{-7}$	3.130 × $10{}^{-7}$	5.560 × $10{}^{-7}$	2.900 × $10{}^{-7}$	7.020 × $10{}^{-7}$
		STD	3.520 × $10{}^{-7}$	1.060 × $10{}^{-6}$	3.500 × $10{}^{-7}$	3.660 × $10{}^{-7}$	4.300 × $10{}^{-7}$	3.310 × $10{}^{-7}$	1.630 × $10{}^{-7}$	2.880 × $10{}^{-7}$	4.810 × $10{}^{-7}$	2.140 × $10{}^{-7}$	6.130 × $10{}^{-7}$
	4	MIN	4.950 × $10{}^{-9}$	3.290 × $10{}^{-8}$	4.820 × $10{}^{-9}$	7.000 × $10{}^{-9}$	9.740 × $10{}^{-9}$	5.550 × $10{}^{-9}$	6.610 × $10{}^{-10}$	5.950 × $10{}^{-9}$	7.250 × $10{}^{-9}$	1.110 × $10{}^{-8}$	1.510 × $10{}^{-8}$
		MAX	2.130 × $10{}^{-6}$	5.760 × $10{}^{-6}$	1.100 × $10{}^{-6}$	2.210 × $10{}^{-6}$	1.770 × $10{}^{-6}$	1.330 × $10{}^{-6}$	7.350 × $10{}^{-7}$	1.550 × $10{}^{-6}$	1.960 × $10{}^{-6}$	1.080 × $10{}^{-6}$	2.460 × $10{}^{-6}$
		MEAN	3.380 × $10{}^{-7}$	1.310 × $10{}^{-6}$	2.580 × $10{}^{-7}$	3.950 × $10{}^{-7}$	4.720 × $10{}^{-7}$	2.980 × $10{}^{-7}$	1.560 × $10{}^{-7}$	3.140 × $10{}^{-7}$	5.280 × $10{}^{-7}$	2.490 × $10{}^{-7}$	6.890 × $10{}^{-7}$
		STD	3.780 × $10{}^{-7}$	1.160 × $10{}^{-6}$	2.570 × $10{}^{-7}$	3.360 × $10{}^{-7}$	4.530 × $10{}^{-7}$	2.830 × $10{}^{-7}$	1.240 × $10{}^{-7}$	3.090 × $10{}^{-7}$	4.520 × $10{}^{-7}$	2.080 × $10{}^{-7}$	5.930 × $10{}^{-7}$

**Table 3:** Absolute errors of problem 3 for different inputs in terms of minimum, maximum, mean and standard deviation.

	Case	Mode	Absolute Errors for Inputs “η”										
			η = 0	η = 0.1	η = 0.2	η = 0.3	η = 0.4	η = 0.5	η = 0.6	η = 0.7	η = 0.8	η = 0.9	η = 1.0
ρ	1	MIN	6.710 × $10{}^{-9}$	3.990 × $10{}^{-8}$	1.180 × $10{}^{-9}$	2.350 × $10{}^{-8}$	1.360 × $10{}^{-8}$	1.040 × $10{}^{-8}$	3.080 × $10{}^{-9}$	3.450 × $10{}^{-9}$	1.860 × $10{}^{-8}$	2.720 × $10{}^{-8}$	3.100 × $10{}^{-8}$
		MAX	1.93 × $10{}^{-6}$	6.98 × $10{}^{-6}$	1.84 × $10{}^{-6}$	3.28 × $10{}^{-6}$	2.22 × $10{}^{-6}$	1.94 × $10{}^{-6}$	1.35 × $10{}^{-6}$	2.17 × $10{}^{-6}$	2.72 × $10{}^{-6}$	9.43 × $10{}^{-7}$	4.26 × $10{}^{-6}$
		MEAN	3.84 × $10{}^{-7}$	1.39 × $10{}^{-6}$	3.16 × $10{}^{-7}$	4.10 × $10{}^{-7}$	5.05 × $10{}^{-7}$	3.46 × $10{}^{-7}$	1.75 × $10{}^{-7}$	3.13 × $10{}^{-7}$	5.56 × $10{}^{-7}$	2.90 × $10{}^{-7}$	7.02 × $10{}^{-7}$
		STD	3.52 × $10{}^{-7}$	1.06 × $10{}^{-6}$	3.50 × $10{}^{-7}$	3.66 × $10{}^{-7}$	4.30 × $10{}^{-7}$	3.31 × $10{}^{-7}$	1.63 × $10{}^{-7}$	2.88 × $10{}^{-7}$	4.81 × $10{}^{-7}$	2.14 × $10{}^{-7}$	6.13 × $10{}^{-7}$
	2	MIN	1.73 × $10{}^{-9}$	1.97 × $10{}^{-8}$	2.69 × $10{}^{-10}$	1.16 × $10{}^{-8}$	1.65 × $10{}^{-9}$	9.28 × $10{}^{-10}$	4.86 × $10{}^{-9}$	1.21 × $10{}^{-9}$	7.76 × $10{}^{-9}$	5.11 × $10{}^{-9}$	9.06 × $10{}^{-9}$
		MAX	2.28 × $10{}^{-6}$	6.20 × $10{}^{-6}$	1.05 × $10{}^{-6}$	1.25 × $10{}^{-6}$	2.97 × $10{}^{-6}$	1.23 × $10{}^{-6}$	3.14 × $10{}^{-7}$	1.81 × $10{}^{-6}$	2.73 × $10{}^{-6}$	8.44 × $10{}^{-7}$	3.57 × $10{}^{-6}$
		MEAN	2.53 × $10{}^{-7}$	6.61 × $10{}^{-7}$	1.48 × $10{}^{-7}$	1.37 × $10{}^{-7}$	2.76 × $10{}^{-7}$	1.84 × $10{}^{-7}$	5.56 × $10{}^{-8}$	1.44 × $10{}^{-7}$	2.97 × $10{}^{-7}$	1.28 × $10{}^{-7}$	3.53 × $10{}^{-7}$
		STD	3.65 × $10{}^{-7}$	8.94 × $10{}^{-7}$	1.70 × $10{}^{-7}$	1.74 × $10{}^{-7}$	4.15 × $10{}^{-7}$	2.04 × $10{}^{-7}$	5.35 × $10{}^{-8}$	2.41 × $10{}^{-7}$	3.82 × $10{}^{-7}$	1.21 × $10{}^{-7}$	4.87 × $10{}^{-7}$
	3	MIN	7.61 × $10{}^{-9}$	1.82 × $10{}^{-8}$	5.97 × $10{}^{-9}$	2.16 × $10{}^{-9}$	5.27 × $10{}^{-9}$	8.92 × $10{}^{-9}$	4.97 × $10{}^{-10}$	1.68 × $10{}^{-9}$	3.02 × $10{}^{-9}$	3.45 × $10{}^{-9}$	1.19 × $10{}^{-8}$
		MAX	1.13 × $10{}^{-5}$	2.22 × $10{}^{-6}$	7.58 × $10{}^{-6}$	6.43 × $10{}^{-6}$	1.84 × $10{}^{-6}$	8.41 × $10{}^{-7}$	4.68 × $10{}^{-6}$	8.03 × $10{}^{-6}$	5.34 × $10{}^{-6}$	3.54 × $10{}^{-7}$	1.51 × $10{}^{-5}$
		MEAN	3.20 × $10{}^{-7}$	3.45 × $10{}^{-7}$	2.03 × $10{}^{-7}$	1.34 × $10{}^{-7}$	1.70 × $10{}^{-7}$	1.11 × $10{}^{-7}$	8.67 × $10{}^{-8}$	1.81 × $10{}^{-7}$	2.20 × $10{}^{-7}$	6.19 × $10{}^{-8}$	3.76 × $10{}^{-7}$
		STD	1.15 × $10{}^{-6}$	3.54 × $10{}^{-7}$	7.67 × $10{}^{-7}$	6.53 × $10{}^{-7}$	2.22 × $10{}^{-7}$	1.22 × $10{}^{-7}$	4.71 × $10{}^{-7}$	8.09 × $10{}^{-7}$	5.45 × $10{}^{-7}$	5.98 × $10{}^{-8}$	1.52 × $10{}^{-6}$
	4	MIN	3.00 × $10{}^{-10}$	1.25 × $10{}^{-10}$	4.10 × $10{}^{-10}$	1.95 × $10{}^{-11}$	1.04 × $10{}^{-10}$	9.47 × $10{}^{-10}$	1.75 × $10{}^{-10}$	2.04 × $10{}^{-10}$	1.25 × $10{}^{-9}$	2.17 × $10{}^{-10}$	1.07 × $10{}^{-9}$
		MAX	3.95 × $10{}^{-6}$	3.65 × $10{}^{-7}$	1.41 × $10{}^{-6}$	1.46 × $10{}^{-6}$	1.49 × $10{}^{-6}$	1.49 × $10{}^{-6}$	9.68 × $10{}^{-7}$	1.28 × $10{}^{-6}$	1.08 × $10{}^{-6}$	7.81 × $10{}^{-7}$	3.27 × $10{}^{-6}$
		MEAN	2.31 × $10{}^{-7}$	4.36 × $10{}^{-8}$	1.16 × $10{}^{-7}$	9.53 × $10{}^{-8}$	4.89 × $10{}^{-8}$	3.51 × $10{}^{-8}$	6.13 × $10{}^{-8}$	9.36 × $10{}^{-8}$	7.80 × $10{}^{-8}$	2.08 × $10{}^{-8}$	1.99 × $10{}^{-7}$
		STD	5.97 × $10{}^{-7}$	5.45 × $10{}^{-8}$	2.38 × $10{}^{-7}$	2.50 × $10{}^{-7}$	1.66 × $10{}^{-7}$	1.49 × $10{}^{-7}$	1.41 × $10{}^{-7}$	2.27 × $10{}^{-7}$	1.77 × $10{}^{-7}$	7.93 × $10{}^{-8}$	4.97 × $10{}^{-7}$
E	1	MIN	6.71 × $10{}^{-9}$	3.99 × $10{}^{-8}$	1.18 × $10{}^{-9}$	2.35 × $10{}^{-8}$	1.36 × $10{}^{-8}$	1.04 × $10{}^{-8}$	3.08 × $10{}^{-9}$	3.45 × $10{}^{-9}$	1.86 × $10{}^{-8}$	2.72 × $10{}^{-8}$	3.10 × $10{}^{-8}$
		MAX	1.93 × $10{}^{-6}$	6.98 × $10{}^{-6}$	1.84 × $10{}^{-6}$	3.28 × $10{}^{-6}$	2.22 × $10{}^{-6}$	1.94 × $10{}^{-6}$	1.35 × $10{}^{-6}$	2.17 × $10{}^{-6}$	2.72 × $10{}^{-6}$	9.43 × $10{}^{-7}$	4.26 × $10{}^{-6}$
		MEAN	3.84 × $10{}^{-7}$	1.39 × $10{}^{-6}$	3.16 × $10{}^{-7}$	4.10 × $10{}^{-7}$	5.05 × $10{}^{-7}$	3.46 × $10{}^{-7}$	1.75 × $10{}^{-7}$	3.13 × $10{}^{-7}$	5.56 × $10{}^{-7}$	2.90 × $10{}^{-7}$	7.02 × $10{}^{-7}$
		STD	3.84 × $10{}^{-7}$	1.39 × $10{}^{-6}$	3.16 × $10{}^{-7}$	4.10 × $10{}^{-7}$	5.05 × $10{}^{-7}$	3.46 × $10{}^{-7}$	1.75 × $10{}^{-7}$	3.13 × $10{}^{-7}$	5.56 × $10{}^{-7}$	2.90 × $10{}^{-7}$	7.02 × $10{}^{-7}$
	2	MIN	1.73 × $10{}^{-9}$	1.97 × $10{}^{-8}$	2.69 × $10{}^{-10}$	1.16 × $10{}^{-8}$	1.65 × $10{}^{-9}$	9.28 × $10{}^{-10}$	4.86 × $10{}^{-9}$	1.21 × $10{}^{-9}$	7.76 × $10{}^{-9}$	5.11 × $10{}^{-9}$	9.06 × $10{}^{-9}$
		MAX	2.28 × $10{}^{-6}$	6.20 × $10{}^{-6}$	1.05 × $10{}^{-6}$	1.25 × $10{}^{-6}$	2.97 × $10{}^{-6}$	1.23 × $10{}^{-6}$	3.14 × $10{}^{-7}$	1.81 × $10{}^{-6}$	2.73 × $10{}^{-6}$	8.44 × $10{}^{-7}$	3.57 × $10{}^{-6}$
		MEAN	2.53 × $10{}^{-7}$	6.61 × $10{}^{-7}$	1.48 × $10{}^{-7}$	1.37 × $10{}^{-7}$	2.76 × $10{}^{-7}$	1.84 × $10{}^{-7}$	5.56 × $10{}^{-8}$	1.44 × $10{}^{-7}$	2.97 × $10{}^{-7}$	1.28 × $10{}^{-7}$	3.53 × $10{}^{-7}$
		STD	3.65 × $10{}^{-7}$	8.94 × $10{}^{-7}$	1.70 × $10{}^{-7}$	1.74 × $10{}^{-7}$	4.15 × $10{}^{-7}$	2.04 × $10{}^{-7}$	5.35 × $10{}^{-8}$	2.41 × $10{}^{-7}$	3.82 × $10{}^{-7}$	1.21 × $10{}^{-7}$	4.87 × $10{}^{-7}$
	3	MIN	7.61 × $10{}^{-9}$	1.82 × $10{}^{-8}$	5.97 × $10{}^{-9}$	2.16 × $10{}^{-9}$	5.27 × $10{}^{-9}$	8.92 × $10{}^{-9}$	4.97 × $10{}^{-10}$	1.68 × $10{}^{-9}$	3.02 × $10{}^{-9}$	3.45 × $10{}^{-9}$	1.19 × $10{}^{-8}$
		MAX	1.13 × $10{}^{-5}$	2.22 × $10{}^{-6}$	7.58 × $10{}^{-6}$	6.43 × $10{}^{-6}$	1.84 × $10{}^{-6}$	8.41 × $10{}^{-7}$	4.68 × $10{}^{-6}$	8.03 × $10{}^{-6}$	5.34 × $10{}^{-6}$	3.54 × $10{}^{-7}$	1.51 × $10{}^{-5}$
		MEAN	3.20 × $10{}^{-7}$	3.45 × $10{}^{-7}$	2.03 × $10{}^{-7}$	1.34 × $10{}^{-7}$	1.70 × $10{}^{-7}$	1.11 × $10{}^{-7}$	8.67 × $10{}^{-8}$	1.81 × $10{}^{-7}$	2.20 × $10{}^{-7}$	6.19 × $10{}^{-8}$	3.76 × $10{}^{-7}$
		STD	1.15 × $10{}^{-6}$	3.54 × $10{}^{-7}$	7.67 × $10{}^{-7}$	6.53 × $10{}^{-7}$	2.22 × $10{}^{-7}$	1.22 × $10{}^{-7}$	4.71 × $10{}^{-7}$	8.09 × $10{}^{-7}$	5.45 × $10{}^{-7}$	5.98 × $10{}^{-8}$	1.52 × $10{}^{-6}$
	4	MIN	3.00 × $10{}^{-10}$	1.25 × $10{}^{-10}$	4.10 × $10{}^{-10}$	1.95 × $10{}^{-11}$	1.04 × $10{}^{-10}$	9.47 × $10{}^{-10}$	1.75 × $10{}^{-10}$	2.04 × $10{}^{-10}$	1.25 × $10{}^{-9}$	2.17 × $10{}^{-10}$	1.07 × $10{}^{-9}$
		MAX	3.95 × $10{}^{-6}$	3.65 × $10{}^{-7}$	1.41 × $10{}^{-6}$	1.46 × $10{}^{-6}$	1.49 × $10{}^{-6}$	1.49 × $10{}^{-6}$	9.68 × $10{}^{-7}$	1.28 × $10{}^{-6}$	1.08 × $10{}^{-6}$	7.81 × $10{}^{-7}$	3.27 × $10{}^{-6}$
		MEAN	2.31 × $10{}^{-7}$	4.36 × $10{}^{-8}$	1.16 × $10{}^{-7}$	9.53 × $10{}^{-8}$	4.89 × $10{}^{-8}$	3.51 × $10{}^{-8}$	6.13 × $10{}^{-8}$	9.36 × $10{}^{-8}$	7.80 × $10{}^{-8}$	2.08 × $10{}^{-8}$	1.99 × $10{}^{-7}$
		STD	5.97 × $10{}^{-7}$	5.45 × $10{}^{-8}$	2.38 × $10{}^{-7}$	2.50 × $10{}^{-7}$	1.66 × $10{}^{-7}$	1.49 × $10{}^{-7}$	1.41 × $10{}^{-7}$	2.27 × $10{}^{-7}$	1.77 × $10{}^{-7}$	7.93 × $10{}^{-8}$	4.97 × $10{}^{-7}$
ϕ	1	MIN	6.71 × $10{}^{-9}$	3.99 × $10{}^{-8}$	1.18 × $10{}^{-9}$	2.35 × $10{}^{-8}$	1.36 × $10{}^{-8}$	1.04 × $10{}^{-8}$	3.08 × $10{}^{-9}$	3.45 × $10{}^{-9}$	1.86 × $10{}^{-8}$	2.72 × $10{}^{-8}$	3.10 × $10{}^{-8}$
		MAX	1.93 × $10{}^{-6}$	6.98 × $10{}^{-6}$	1.84 × $10{}^{-6}$	3.28 × $10{}^{-6}$	2.22 × $10{}^{-6}$	1.94 × $10{}^{-6}$	1.35 × $10{}^{-6}$	2.17 × $10{}^{-6}$	2.72 × $10{}^{-6}$	9.43 × $10{}^{-7}$	4.26 × $10{}^{-6}$
		MEAN	3.84 × $10{}^{-7}$	1.39 × $10{}^{-6}$	3.16 × $10{}^{-7}$	4.10 × $10{}^{-7}$	5.05 × $10{}^{-7}$	3.46 × $10{}^{-7}$	1.75 × $10{}^{-7}$	3.13 × $10{}^{-7}$	5.56 × $10{}^{-7}$	2.90 × $10{}^{-7}$	7.02 × $10{}^{-7}$
		STD	3.52 × $10{}^{-7}$	1.06 × $10{}^{-6}$	3.50 × $10{}^{-7}$	3.66 × $10{}^{-7}$	4.30 × $10{}^{-7}$	3.31 × $10{}^{-7}$	1.63 × $10{}^{-7}$	2.88 × $10{}^{-7}$	4.81 × $10{}^{-7}$	2.14 × $10{}^{-7}$	6.13 × $10{}^{-7}$
	2	MIN	1.73 × $10{}^{-9}$	1.97 × $10{}^{-8}$	2.69 × $10{}^{-10}$	1.16 × $10{}^{-8}$	1.65 × $10{}^{-9}$	9.28 × $10{}^{-10}$	4.86 × $10{}^{-9}$	1.21 × $10{}^{-9}$	7.76 × $10{}^{-9}$	5.11 × $10{}^{-9}$	9.06 × $10{}^{-9}$
		MAX	2.28 × $10{}^{-6}$	6.20 × $10{}^{-6}$	1.05 × $10{}^{-6}$	1.25 × $10{}^{-6}$	2.97 × $10{}^{-6}$	1.23 × $10{}^{-6}$	3.14 × $10{}^{-7}$	1.81 × $10{}^{-6}$	2.73 × $10{}^{-6}$	8.44 × $10{}^{-7}$	3.57 × $10{}^{-6}$
		MEAN	2.53 × $10{}^{-7}$	6.61 × $10{}^{-7}$	1.48 × $10{}^{-7}$	1.37 × $10{}^{-7}$	2.76 × $10{}^{-7}$	1.84 × $10{}^{-7}$	5.56 × $10{}^{-8}$	1.44 × $10{}^{-7}$	2.97 × $10{}^{-7}$	1.28 × $10{}^{-7}$	3.53 × $10{}^{-7}$
		STD	3.65 × $10{}^{-7}$	8.94 × $10{}^{-7}$	1.70 × $10{}^{-7}$	1.74 × $10{}^{-7}$	4.15 × $10{}^{-7}$	2.04 × $10{}^{-7}$	5.35 × $10{}^{-8}$	2.41 × $10{}^{-7}$	3.82 × $10{}^{-7}$	1.21 × $10{}^{-7}$	4.87 × $10{}^{-7}$
	3	MIN	7.61 × $10{}^{-9}$	1.82 × $10{}^{-8}$	5.97 × $10{}^{-9}$	2.16 × $10{}^{-9}$	5.27 × $10{}^{-9}$	8.92 × $10{}^{-9}$	4.97 × $10{}^{-10}$	1.68 × $10{}^{-9}$	3.02 × $10{}^{-9}$	3.45 × $10{}^{-9}$	1.19 × $10{}^{-8}$
		MAX	1.13 × $10{}^{-5}$	2.22 × $10{}^{-6}$	7.58 × $10{}^{-6}$	6.43 × $10{}^{-6}$	1.84 × $10{}^{-6}$	8.41 × $10{}^{-7}$	4.68 × $10{}^{-6}$	8.03 × $10{}^{-6}$	5.34 × $10{}^{-6}$	3.54 × $10{}^{-7}$	1.51 × $10{}^{-5}$
		MEAN	3.20 × $10{}^{-7}$	3.45 × $10{}^{-7}$	2.03 × $10{}^{-7}$	1.34 × $10{}^{-7}$	1.70 × $10{}^{-7}$	1.11 × $10{}^{-7}$	8.67 × $10{}^{-8}$	1.81 × $10{}^{-7}$	2.20 × $10{}^{-7}$	6.19 × $10{}^{-8}$	3.76 × $10{}^{-7}$
		STD	1.15 × $10{}^{-6}$	3.54 × $10{}^{-7}$	7.67 × $10{}^{-7}$	6.53 × $10{}^{-7}$	2.22 × $10{}^{-7}$	1.22 × $10{}^{-7}$	4.71E -07	8.09 × $10{}^{-7}$	5.45 × $10{}^{-7}$	5.98 × $10{}^{-8}$	1.52 × $10{}^{-6}$
	4	MIN	3.00 × $10{}^{-10}$	1.25 × $10{}^{-10}$	4.10 × $10{}^{-10}$	1.95 × $10{}^{-11}$	1.04 × $10{}^{-10}$	9.47 × $10{}^{-10}$	1.75 × $10{}^{-10}$	2.04 × $10{}^{-10}$	1.25 × $10{}^{-9}$	2.17 × $10{}^{-10}$	1.07 × $10{}^{-9}$
		MAX	3.95 × $10{}^{-6}$	3.65 × $10{}^{-7}$	1.41 × $10{}^{-6}$	1.46 × $10{}^{-6}$	1.49 × $10{}^{-6}$	1.49 × $10{}^{-6}$	9.68 × $10{}^{-7}$	1.28 × $10{}^{-6}$	1.08 × $10{}^{-6}$	7.81 × $10{}^{-7}$	3.27 × $10{}^{-6}$
		MEAN	2.31 × $10{}^{-7}$	4.36 × $10{}^{-8}$	1.16 × $10{}^{-7}$	9.53 × $10{}^{-8}$	4.89 × $10{}^{-8}$	3.51 × $10{}^{-8}$	6.13 × $10{}^{-8}$	9.36 × $10{}^{-8}$	7.80 × $10{}^{-8}$	2.08 × $10{}^{-8}$	1.99 × $10{}^{-7}$
		STD	5.97 × $10{}^{-7}$	5.45 × $10{}^{-8}$	2.38 × $10{}^{-7}$	2.50 × $10{}^{-7}$	1.66 × $10{}^{-7}$	1.49 × $10{}^{-7}$	1.41 × $10{}^{-7}$	2.27 × $10{}^{-7}$	1.77 × $10{}^{-7}$	7.93 × $10{}^{-8}$	4.97 × $10{}^{-7}$

**Table 4:** Analysis of ANN-SCA-SQP by variation of number of neurons.

No. of Neurons	Variable	Absolute Errors for Inputs η										
		η = 0	η = 0.1	η = 0.2	η = 0.3	η = 0.4	η = 0.5	η = 0.6	η = 0.7	η = 0.8	η = 0.9	η = 1.0
9	ρ	1.603 × $10{}^{-1}$	1.562 × $10{}^{-1}$	1.526 × $10{}^{-1}$	1.495 × $10{}^{-1}$	1.469 × $10{}^{-1}$	1.447 × $10{}^{-1}$	1.430 × $10{}^{-1}$	1.417 × $10{}^{-2}$	1.4093 × $10{}^{-1}$	1.405 × $10{}^{-1}$	1.404 × $10{}^{-1}$
	E	1.961 × $10{}^{-1}$	1.520 × $10{}^{-1}$	1.195 × $10{}^{-1}$	9.826 × $10{}^{-2}$	8.640 × $10{}^{-2}$	8.099 × $10{}^{-2}$	7.810 × $10{}^{-2}$	7.356 × $10{}^{-2}$	6.363 × $10{}^{-2}$	4.557 × $10{}^{-2}$	1.792 × $10{}^{-2}$
	ϕ	1.9989 × $10{}^{-2}$	6.68 × $10{}^{-3}$	2.861 × $10{}^{-3}$	1.0292 × $10{}^{-2}$	1.729 × $10{}^{-2}$	2.5269 × $10{}^{-2}$	3.4953 × $10{}^{-2}$	4.590 × $10{}^{-2}$	5.617 × $10{}^{-2}$	6.2283 × $10{}^{-2}$	5.9759 × $10{}^{-2}$
27	ρ	3.57 × $10{}^{-5}$	8.70 × $10{}^{-5}$	1.16 × $10{}^{-4}$	7.95 × $10{}^{-5}$	1.47 × $10{}^{-6}$	9.46 × $10{}^{-5}$	1.88 × $10{}^{-4}$	2.59 × $10{}^{-4}$	2.91 × $10{}^{-4}$	2.68 × $10{}^{-4}$	1.76 × $10{}^{-4}$
	E	6.24 × $10{}^{-7}$	1.06 × $10{}^{-5}$	1.49 × $10{}^{-6}$	1.67 × $10{}^{-5}$	3.53 × $10{}^{-5}$	4.81 × $10{}^{-5}$	5.26 × $10{}^{-5}$	5.04 × $10{}^{-5}$	4.79 × $10{}^{-5}$	5.66 × $10{}^{-5}$	9.29 × $10{}^{-5}$
	ϕ	2.12 × $10{}^{-6}$	1.82 × $10{}^{-4}$	4.37 × $10{}^{-4}$	4.36 × $10{}^{-4}$	2.29 × $10{}^{-4}$	2.10 × $10{}^{-5}$	1.600 × $10{}^{-4}$	1.05 × $10{}^{-4}$	1.18 × $10{}^{-4}$	3.61 × $10{}^{-4}$	3.34 × $10{}^{-4}$
45	ρ	5.30 × $10{}^{-7}$	1.58 × $10{}^{-7}$	1.41 × $10{}^{-7}$	1.40 × $10{}^{-7}$	1.54 × $10{}^{-7}$	1.71 × $10{}^{-7}$	1.36 × $10{}^{-7}$	1.20 × $10{}^{-7}$	1.28 × $10{}^{-7}$	1.51 × $10{}^{-7}$	3.57 × $10{}^{-7}$
	E	1.59 × $10{}^{-8}$	3.54 × $10{}^{-9}$	4.85 × $10{}^{-9}$	6.65 × $10{}^{-9}$	2.90 × $10{}^{-9}$	6.18 × $10{}^{-10}$	1.98 × $10{}^{-9}$	2.49 × $10{}^{-9}$	1.36 × $10{}^{-9}$	1.03 × $10{}^{-9}$	3.89 × $10{}^{-9}$
	ϕ	1.59 × $10{}^{-8}$	3.54 × $10{}^{-9}$	4.85 × $10{}^{-9}$	6.65 × $10{}^{-9}$	2.90 × $10{}^{-9}$	6.18 × $10{}^{-10}$	1.98 × $10{}^{-9}$	2.49 × $10{}^{-9}$	1.36 × $10{}^{-9}$	1.03 × $10{}^{-9}$	3.89 × $10{}^{-9}$
90	ρ	4.85 × $10{}^{-7}$	2.81 × $10{}^{-6}$	2.73 × $10{}^{-6}$	1.02 × $10{}^{-5}$	1.55 × $10{}^{-5}$	1.69 × $10{}^{-5}$	1.43 × $10{}^{-5}$	8.96 × $10{}^{-6}$	3.28 × $10{}^{-6}$	5.59 × $10{}^{-8}$	2.36 × $10{}^{-6}$
	E	6.04 × $10{}^{-7}$	3.82 × $10{}^{-6}$	4.86 × $10{}^{-6}$	1.49 × $10{}^{-5}$	2.05 × $10{}^{-5}$	1.94 × $10{}^{-5}$	1.24 × $10{}^{-5}$	2.23 × $10{}^{-6}$	7.29 × $10{}^{-6}$	1.17 × $10{}^{-5}$	6.53 × $10{}^{-6}$
	ϕ	5.83 × $10{}^{-7}$	3.12 × $10{}^{-5}$	6.59 × $10{}^{-5}$	4.84 × $10{}^{-5}$	8.70 × $10{}^{-6}$	8.68 × $10{}^{-6}$	1.22 × $10{}^{-5}$	4.80 × $10{}^{-5}$	5.69 × $10{}^{-5}$	2.06 × $10{}^{-5}$	3.70 × $10{}^{-6}$

**Table 5:** Analysis of ANN-SCA-SQP by variation of population size.

Population Size	Variable	Absolute Errors for Inputs η										
		η = 0	η = 0.1	η = 0.2	η = 0.3	η = 0.4	η = 0.5	η = 0.6	η = 0.7	η = 0.8	η = 0.9	η = 1.0
20	ρ	1.79 × $10{}^{-6}$	3.64 × $10{}^{-5}$	3.84 × $10{}^{-5}$	1.84 × $10{}^{-5}$	1.23 × $10{}^{-5}$	4.44 × $10{}^{-5}$	7.12 × $10{}^{-5}$	8.77 × $10{}^{-5}$	9.05 × $10{}^{-5}$	7.79 × $10{}^{-5}$	4.91 × $10{}^{-5}$
	E	3.24 × $10{}^{-7}$	3.60 × $10{}^{-6}$	1.25 × $10{}^{-5}$	1.82 × $10{}^{-5}$	1.75 × $10{}^{-5}$	1.13 × $10{}^{-5}$	1.97 × $10{}^{-6}$	7.52 × $10{}^{-6}$	1.52 × $10{}^{-5}$	2.07 × $10{}^{-5}$	2.62 × $10{}^{-5}$
	ϕ	5.44 × $10{}^{-7}$	1.69 × $10{}^{-5}$	1.80 × $10{}^{-5}$	3.73 × $10{}^{-6}$	2.03 × $10{}^{-5}$	1.46 × $10{}^{-5}$	6.95 × $10{}^{-6}$	2.35 × $10{}^{-5}$	1.80 × $10{}^{-5}$	5.32 × $10{}^{-6}$	1.11 × $10{}^{-5}$
30	ρ	5.30 × $10{}^{-7}$	1.58 × $10{}^{-7}$	1.41 × $10{}^{-7}$	1.40 × $10{}^{-7}$	1.54 × $10{}^{-7}$	1.71 × $10{}^{-7}$	1.36 × $10{}^{-7}$	1.20 × $10{}^{-7}$	1.28 × $10{}^{-7}$	1.51 × $10{}^{-7}$	3.57 × $10{}^{-7}$
	E	1.59 × $10{}^{-8}$	3.54 × $10{}^{-9}$	4.85 × $10{}^{-9}$	6.65 × $10{}^{-9}$	2.90 × $10{}^{-9}$	6.18 × $10{}^{-10}$	1.98 × $10{}^{-9}$	2.49 × $10{}^{-9}$	1.36 × $10{}^{-9}$	1.03 × $10{}^{-9}$	3.89 × $10{}^{-9}$
	ϕ	1.59 × $10{}^{-8}$	3.54 × $10{}^{-9}$	4.85 × $10{}^{-9}$	6.65 × $10{}^{-9}$	2.90 × $10{}^{-9}$	6.18 × $10{}^{-10}$	1.98 × $10{}^{-9}$	2.49 × $10{}^{-9}$	1.36 × $10{}^{-9}$	1.03 × $10{}^{-9}$	3.89 × $10{}^{-9}$
40	ρ	1.01 × $10{}^{-6}$	1.01 × $10{}^{-6}$	9.25 × $10{}^{-6}$	1.79 × $10{}^{-5}$	2.22 × $10{}^{-5}$	2.01 × $10{}^{-5}$	1.21 × $10{}^{-5}$	6.92 × $10{}^{-7}$	9.90 × $10{}^{-6}$	1.41 × $10{}^{-5}$	5.25 × $10{}^{-6}$
	E	1.78 × $10{}^{-6}$	9.66 × $10{}^{-6}$	4.78 × $10{}^{-5}$	6.69 × $10{}^{-5}$	5.52 × $10{}^{-5}$	2.45 × $10{}^{-5}$	3.96 × $10{}^{-6}$	1.14 × $10{}^{-5}$	8.18 × $10{}^{-6}$	4.00 × $10{}^{-5}$	4.21 × $10{}^{-5}$
	ϕ	1.47 × $10{}^{-6}$	1.97 × $10{}^{-5}$	9.03 × $10{}^{-5}$	1.15 × $10{}^{-5}$	8.11 × $10{}^{-5}$	2.11 × $10{}^{-5}$	2.41 × $10{}^{-5}$	2.70 × $10{}^{-5}$	1.27 × $10{}^{-5}$	6.27 × $10{}^{-5}$	6.14 × $10{}^{-5}$

**Table 6:** Comparison of ANN-SCA-SQP with other techniques.

Algorithm	Varaible	Absolute Errors for Inputs η										
		η = 0	η = 0.1	η = 0.2	η = 0.3	η = 0.4	η = 0.5	η = 0.6	η = 0.7	η = 0.8	η = 0.9	η = 1.0
ANN-SCA-SQP	ρ	5.30 × $10{}^{-7}$	1.58 × $10{}^{-7}$	1.41 × $10{}^{-7}$	1.40 × $10{}^{-7}$	1.54 × $10{}^{-7}$	1.71 × $10{}^{-7}$	1.36 × $10{}^{-7}$	1.20 × $10{}^{-7}$	1.28 × $10{}^{-7}$	1.51 × $10{}^{-7}$	3.57 × $10{}^{-7}$
	E	1.59 × $10{}^{-8}$	3.54 × $10{}^{-9}$	4.85 × $10{}^{-9}$	6.65 × $10{}^{-9}$	2.90 × $10{}^{-9}$	6.18 × $10{}^{-10}$	1.98 × $10{}^{-9}$	2.49 × $10{}^{-9}$	1.36 × $10{}^{-9}$	1.03 × $10{}^{-9}$	3.89 × $10{}^{-9}$
	ϕ	1.59 × $10{}^{-8}$	3.54 × $10{}^{-9}$	4.85 × $10{}^{-9}$	6.65 × $10{}^{-9}$	2.90 × $10{}^{-9}$	6.18 × $10{}^{-10}$	1.98 × $10{}^{-9}$	2.49 × $10{}^{-9}$	1.36 × $10{}^{-9}$	1.03 × $10{}^{-9}$	3.89 × $10{}^{-9}$
GA-SQP	ρ	4.408 × $10{}^{-3}$	1.077 × $10{}^{-1}$	1.855 × $10{}^{-1}$	2.450 × $10{}^{-1}$	2.912 × $10{}^{-1}$	3.280 × $10{}^{-1}$	3.580 × $10{}^{-1}$	3.830 × $10{}^{-1}$	4.045 × $10{}^{-1}$	4.232 × $10{}^{-1}$	4.399 × $10{}^{-1}$
	E	1.074 × $10{}^{-3}$	4.796 × $10{}^{-3}$	2.011 × $10{}^{-2}$	4.234 × $10{}^{-2}$	6.968 × $10{}^{-2}$	1.008 × $10{}^{-1}$	1.351 × $10{}^{-1}$	1.719 × $10{}^{-1}$	2.109 × $10{}^{-1}$	2.522 × $10{}^{-1}$	2.956 × $10{}^{-1}$
	ϕ	1.43 × $10{}^{-5}$	5.13 × $10{}^{-4}$	3.93 × $10{}^{-4}$	2.168 × $10{}^{-3}$	7.184 × $10{}^{-3}$	1.604 × $10{}^{-2}$	2.889 × $10{}^{-2}$	4.558 × $10{}^{-2}$	6.579 × $10{}^{-2}$	8.908 × $10{}^{-2}$	1.151 × $10{}^{-1}$
PSO-SQP	ρ	1.09 × $10{}^{-6}$	3.25 × $10{}^{-5}$	1.12 × $10{}^{-4}$	1.28 × $10{}^{-4}$	1.00 × $10{}^{-4}$	7.12 × $10{}^{-5}$	5.95 × $10{}^{-5}$	5.85 × $10{}^{-5}$	5.96 × $10{}^{-5}$	7.02 × $10{}^{-5}$	8.00 × $10{}^{-5}$
	E	9.80 × $10{}^{-6}$	1.34 × $10{}^{-4}$	2.43 × $10{}^{-4}$	2.16 × $10{}^{-4}$	1.67 × $10{}^{-4}$	1.44 × $10{}^{-4}$	1.39 × $10{}^{-4}$	1.60 × $10{}^{-4}$	2.14 × $10{}^{-4}$	2.42 × $10{}^{-4}$	1.98 × $10{}^{-4}$
	ϕ	9.79 × $10{}^{-7}$	2.14 × $10{}^{-5}$	1.18 × $10{}^{-4}$	1.87 × $10{}^{-4}$	1.84 × $10{}^{-4}$	1.19 × $10{}^{-4}$	4.46 × $10{}^{-5}$	3.42 × $10{}^{-5}$	1.34 × $10{}^{-4}$	3.03 × $10{}^{-4}$	3.47 × $10{}^{-4}$

**Table 7:** Values of performance matrices in term of mean and standard deviation.

Problem	Index	Mode	MEAN				STD			
			Case 1	Case 2	Case 3	Case 4	Case 1	Case 2	Case 3	Case 4
		MAD	2.210 × $10{}^{-5}$	2.120 × $10{}^{-5}$	1.68 × $10{}^{-4}$	1.194 × $10{}^{-3}$	2.570 × $10{}^{-5}$	1.330 × $10{}^{-5}$	2.59 × $10{}^{-4}$	2.646 × $10{}^{-3}$
	ρ	RMSE	2.700 × $10{}^{-5}$	2.750 × $10{}^{-5}$	1.989 × $10{}^{-4}$	1.425 × $10{}^{-3}$	3.030 × $10{}^{-5}$	1.720 × $10{}^{-5}$	2.90 × $10{}^{-4}$	3.031 × $10{}^{-3}$
		ENSE	5.990 × $10{}^{-6}$	3.200 × $10{}^{-7}$	3.320 × $10{}^{-5}$	2.249 × $10{}^{-3}$	1.200 × $10{}^{-5}$	5.300 × $10{}^{-7}$	1.66 × $10{}^{-4}$	1.498 × $10{}^{-2}$
		MAD	1.830 × $10{}^{-5}$	4.680 × $10{}^{-5}$	1.481 × $10{}^{-4}$	7.475 × $10{}^{-4}$	1.430 × $10{}^{-5}$	3.010 × $10{}^{-5}$	1.71 × $10{}^{-4}$	1.745 × $10{}^{-3}$
1	E	RMSE	2.300 × $10{}^{-5}$	5.730 × $10{}^{-5}$	1.71 × $10{}^{-4}$	8.549 × $10{}^{-4}$	1.870 × $10{}^{-5}$	3.720 × $10{}^{-5}$	1.94 × $10{}^{-4}$	1.971 × $10{}^{-3}$
		ENSE	3.520 × $10{}^{-8}$	5.050 × $10{}^{-7}$	1.690 × $10{}^{-5}$	1.914 × $10{}^{-3}$	7.410 × $10{}^{-8}$	6.040 × $10{}^{-7}$	6.160 × $10{}^{-5}$	1.353 × $10{}^{-2}$
		MAD	2.080 × $10{}^{-5}$	2.680 × $10{}^{-5}$	8.290 × $10{}^{-5}$	3.404 × $10{}^{-4}$	1.090 × $10{}^{-5}$	1.220 × $10{}^{-5}$	8.130 × $10{}^{-5}$	6.74 × $10{}^{-4}$
	ϕ	RMSE	2.590 × $10{}^{-5}$	3.300 × $10{}^{-5}$	1.017 × $10{}^{-4}$	4.326 × $10{}^{-4}$	1.370 × $10{}^{-5}$	1.490 × $10{}^{-5}$	9.860 × $10{}^{-5}$	8.93 × $10{}^{-4}$
		ENSE	1.690 × $10{}^{-7}$	3.300 × $10{}^{-7}$	1.060 × $10{}^{-5}$	7.492 × $10{}^{-4}$	2.340 × $10{}^{-7}$	4.180 × $10{}^{-7}$	4.670 × $10{}^{-5}$	5.213 × $10{}^{-3}$
		MAD	3.080 × $10{}^{-5}$	2.240 × $10{}^{-5}$	2.140 × $10{}^{-5}$	2.120 × $10{}^{-5}$	2.420 × $10{}^{-5}$	3.400 × $10{}^{-5}$	1.190 × $10{}^{-5}$	1.330 × $10{}^{-5}$
	ρ	RMSE	3.930 × $10{}^{-5}$	2.860 × $10{}^{-5}$	2.760 × $10{}^{-5}$	2.750 × $10{}^{-5}$	3.090 × $10{}^{-5}$	4.130 × $10{}^{-5}$	1.540 × $10{}^{-5}$	1.720 × $10{}^{-5}$
		ENSE	3.000 × $10{}^{-7}$	5.780 × $10{}^{-7}$	2.780 × $10{}^{-7}$	3.200 × $10{}^{-7}$	5.800 × $10{}^{-7}$	4.280 × $10{}^{-6}$	3.410 × $10{}^{-7}$	5.300 × $10{}^{-7}$
		MAD	4.230 × $10{}^{-5}$	4.780 × $10{}^{-5}$	5.080 × $10{}^{-5}$	4.680 × $10{}^{-5}$	4.090 × $10{}^{-5}$	3.540 × $10{}^{-5}$	2.760 × $10{}^{-5}$	3.010 × $10{}^{-5}$
2	E	RMSE	5.130 × $10{}^{-5}$	5.830 × $10{}^{-5}$	6.210 × $10{}^{-5}$	5.730 × $10{}^{-5}$	4.970 × $10{}^{-5}$	4.340 × $10{}^{-5}$	3.380 × $10{}^{-5}$	3.720 × $10{}^{-5}$
		ENSE	1.220 × $10{}^{-6}$	7.740 × $10{}^{-7}$	5.900 × $10{}^{-7}$	5.050 × $10{}^{-7}$	2.930 × $10{}^{-6}$	9.610 × $10{}^{-7}$	5.850 × $10{}^{-7}$	6.040 × $10{}^{-7}$
		MAD	3.560 × $10{}^{-5}$	3.050 × $10{}^{-5}$	3.170 × $10{}^{-5}$	2.680 × $10{}^{-5}$	2.480 × $10{}^{-5}$	1.600 × $10{}^{-5}$	1.700 × $10{}^{-5}$	1.220 × $10{}^{-5}$
	ϕ	RMSE	4.370 × $10{}^{-5}$	3.750 × $10{}^{-5}$	3.920 × $10{}^{-5}$	3.300 × $10{}^{-5}$	2.980 × $10{}^{-5}$	1.910 × $10{}^{-5}$	2.070 × $10{}^{-5}$	1.490 × $10{}^{-5}$
		ENSE	1.460 × $10{}^{-6}$	6.500 × $10{}^{-7}$	5.280 × $10{}^{-7}$	3.300 × $10{}^{-7}$	2.630 × $10{}^{-6}$	9.690 × $10{}^{-7}$	1.120 × $10{}^{-6}$	4.180 × $10{}^{-7}$
		MAD	2.140 × $10{}^{-5}$	1.620 × $10{}^{-5}$	2.020 × $10{}^{-5}$	1.140 × $10{}^{-5}$	1.190 × $10{}^{-5}$	1.750 × $10{}^{-5}$	1.360 × $10{}^{-5}$	1.110 × $10{}^{-5}$
	ρ	RMSE	2.760 × $10{}^{-5}$	2.030 × $10{}^{-5}$	2.510 × $10{}^{-5}$	1.470 × $10{}^{-5}$	1.540 × $10{}^{-5}$	2.140 × $10{}^{-5}$	1.680 × $10{}^{-5}$	1.480 × $10{}^{-5}$
		ENSE	2.780 × $10{}^{-7}$	3.390 × $10{}^{-7}$	5.600 × $10{}^{-7}$	4.770 × $10{}^{-7}$	3.410 × $10{}^{-7}$	1.120 × $10{}^{-6}$	7.670 × $10{}^{-7}$	1.010 × $10{}^{-6}$
		MAD	5.080 × $10{}^{-5}$	3.040 × $10{}^{-5}$	1.470 × $10{}^{-5}$	1.160 × $10{}^{-5}$	2.760 × $10{}^{-5}$	1.330 × $10{}^{-5}$	1.060 × $10{}^{-5}$	1.950 × $10{}^{-5}$
3	E	RMSE	6.210 × $10{}^{-5}$	3.760 × $10{}^{-5}$	1.820 × $10{}^{-5}$	1.460 × $10{}^{-5}$	3.380 × $10{}^{-5}$	1.670 × $10{}^{-5}$	1.310 × $10{}^{-5}$	2.350 × $10{}^{-5}$
		ENSE	5.900 × $10{}^{-7}$	2.930 × $10{}^{-7}$	1.330 × $10{}^{-7}$	5.760 × $10{}^{-7}$	5.850 × $10{}^{-7}$	2.680 × $10{}^{-7}$	2.530 × $10{}^{-7}$	3.880 × $10{}^{-6}$
		MAD	3.170 × $10{}^{-5}$	2.010 × $10{}^{-5}$	1.990 × $10{}^{-5}$	1.530 × $10{}^{-5}$	1.700 × $10{}^{-5}$	1.230 × $10{}^{-5}$	2.810 × $10{}^{-5}$	2.030 × $10{}^{-5}$
	ϕ	RMSE	3.920 × $10{}^{-5}$	2.510 × $10{}^{-5}$	2.500 × $10{}^{-5}$	1.950 × $10{}^{-5}$	2.070 × $10{}^{-5}$	1.530 × $10{}^{-5}$	3.680 × $10{}^{-5}$	2.730 × $10{}^{-5}$
		ENSE	5.280 × $10{}^{-7}$	3.610 × $10{}^{-7}$	1.540 × $10{}^{-6}$	3.680 × $10{}^{-6}$	1.120 × $10{}^{-6}$	7.230 × $10{}^{-7}$	7.930 × $10{}^{-6}$	1.230 × $10{}^{-5}$

**Table 8:** Minimum and mean values of global performance operators.

Problem	Index	Mode	MIN				MEAN			
			Case 1	Case 2	Case 3	Case 4	Case 1	Case 2	Case 3	Case 4
		GMAD	2.100 × $10{}^{-8}$	2.08 × $10{}^{-8}$	1.820 × $10{}^{-7}$	5.250 × $10{}^{-7}$	2.210 × $10{}^{-7}$	2.120 × $10{}^{-7}$	1.680 × $10{}^{-6}$	1.190 × $10{}^{-5}$
	ρ	GRMSE	2.520 × $10{}^{-8}$	3.010 × $10{}^{-8}$	2.250 × $10{}^{-7}$	8.750 × $10{}^{-7}$	2.700 × $10{}^{-7}$	2.75 × $10{}^{-7}$	1.990 × $10{}^{-6}$	1.430 × $10{}^{-5}$
		GENSE	2.180 × $10{}^{-14}$	7.290 × $10{}^{-13}$	1.020 × $10{}^{-12}$	3.62 × $10{}^{-9}$	5.990 × $10{}^{-8}$	3.200 × $10{}^{-9}$	3.320 × $10{}^{-7}$	2.250 × $10{}^{-5}$
		GMAD	2.830 × $10{}^{-8}$	3.670 × $10{}^{-8}$	2.000 × $10{}^{-7}$	8.140 × $10{}^{-7}$	1.830 × $10{}^{-7}$	4.680 × $10{}^{-7}$	1.480 × $10{}^{-6}$	7.480 × $10{}^{-6}$
1	E	GRMSE	3.610 × $10{}^{-8}$	4.250 × $10{}^{-8}$	2.510 × $10{}^{-7}$	1.040 × $10{}^{-6}$	2.300 × $10{}^{-7}$	5.730 × $10{}^{-7}$	1.710 × $10{}^{-6}$	8.550 × $10{}^{-6}$
		GENSE	1.360 × $10{}^{-15}$	2.590 × $10{}^{-12}$	1.080 × $10{}^{-12}$	8.880 × $10{}^{-11}$	3.520 × $10{}^{-10}$	5.050 × $10{}^{-9}$	1.690 × $10{}^{-7}$	1.910 × $10{}^{-5}$
		GMAD	2.920 × $10{}^{-8}$	2.780 × $10{}^{-8}$	1.020 × $10{}^{-7}$	3.470 × $10{}^{-7}$	2.080 × $10{}^{-7}$	2.680 × $10{}^{-7}$	8.290 × $10{}^{-7}$	3.400 × $10{}^{-6}$
	ϕ	GRMSE	3.620 × $10{}^{-8}$	3.470 × $10{}^{-8}$	1.250 × $10{}^{-7}$	4.350 × $10{}^{-7}$	2.590 × $10{}^{-7}$	3.300 × $10{}^{-7}$	1.020 × $10{}^{-6}$	4.330 × $10{}^{-6}$
		GENSE	6.840 × $10{}^{-15}$	4.330 × $10{}^{-15}$	3.250 × $10{}^{-12}$	1.390 × $10{}^{-10}$	1.690 × $10{}^{-9}$	3.300 × $10{}^{-9}$	1.060 × $10{}^{-7}$	7.490 × $10{}^{-6}$
		GMAD	5.110 × $10{}^{-8}$	3.810 × $10{}^{-8}$	4.260 × $10{}^{-6}$	2.080 × $10{}^{-8}$	3.080 × $10{}^{-7}$	2.240 × $10{}^{-7}$	2.140 × $10{}^{-5}$	2.120 × $10{}^{-7}$
	ρ	GRMSE	6.820 × $10{}^{-8}$	5.460 × $10{}^{-8}$	5.410 × $10{}^{-6}$	3.010 × $10{}^{-8}$	3.930 × $10{}^{-7}$	2.860 × $10{}^{-7}$	2.760 × $10{}^{-5}$	2.750 × $10{}^{-7}$
		GENSE	6.840 × $10{}^{-13}$	6.860 × $10{}^{-14}$	1.250 × $10{}^{-12}$	7.290 × $10{}^{-13}$	3.000 × $10{}^{-9}$	5.780 × $10{}^{-9}$	2.780 × $10{}^{-7}$	3.200 × $10{}^{-9}$
		GMAD	4.310 × $10{}^{-8}$	1.970 × $10{}^{-8}$	4.860 × $10{}^{-6}$	3.670 × $10{}^{-8}$	4.230 × $10{}^{-7}$	4.780 × $10{}^{-7}$	5.080 × $10{}^{-5}$	4.680 × $10{}^{-7}$
2	E	GRMSE	6.060 × $10{}^{-8}$	2.230 × $10{}^{-8}$	6.120 × $10{}^{-6}$	4.250 × $10{}^{-8}$	5.130 × $10{}^{-7}$	5.830 × $10{}^{-7}$	6.210 × $10{}^{-5}$	5.730 × $10{}^{-7}$
		GENSE	1.750 × $10{}^{-12}$	7.810 × $10{}^{-14}$	1.450 × $10{}^{-10}$	2.590 × $10{}^{-12}$	1.220 × $10{}^{-8}$	7.740 × $10{}^{-9}$	5.900 × $10{}^{-7}$	5.050 × $10{}^{-9}$
		GMAD	8.300 × $10{}^{-8}$	6.560 × $10{}^{-8}$	3.900 × $10{}^{-6}$	2.780 × $10{}^{-8}$	3.560 × $10{}^{-7}$	3.050 × $10{}^{-7}$	3.170 × $10{}^{-5}$	2.680 × $10{}^{-7}$
	ϕ	GRMSE	1.010 × $10{}^{-7}$	7.650 × $10{}^{-8}$	5.420 × $10{}^{-6}$	3.470 × $10{}^{-8}$	4.370 × $10{}^{-7}$	3.750 × $10{}^{-7}$	3.920 × $10{}^{-5}$	3.300 × $10{}^{-7}$
		GENSE	1.850 × $10{}^{-12}$	1.070 × $10{}^{-12}$	1.330 × $10{}^{-10}$	4.330 × $10{}^{-15}$	1.460 × $10{}^{-8}$	6.500 × $10{}^{-9}$	5.280 × $10{}^{-7}$	3.300 × $10{}^{-9}$
		GMAD	4.260 × $10{}^{-8}$	2.620 × $10{}^{-8}$	1.540 × $10{}^{-8}$	1.150 × $10{}^{-8}$	2.140 × $10{}^{-7}$	1.620 × $10{}^{-7}$	2.020 × $10{}^{-7}$	1.140 × $10{}^{-7}$
	ρ	GRMSE	5.410 × $10{}^{-8}$	3.310 × $10{}^{-8}$	2.060 × $10{}^{-8}$	1.410 × $10{}^{-8}$	2.760 × $10{}^{-7}$	2.030 × $10{}^{-7}$	2.510 × $10{}^{-7}$	1.470 × $10{}^{-7}$
		GENSE	1.250 × $10{}^{-14}$	4.770 × $10{}^{-13}$	5.670 × $10{}^{-14}$	1.560 × $10{}^{-12}$	2.780 × $10{}^{-9}$	3.390 × $10{}^{-9}$	5.600 × $10{}^{-9}$	4.770 × $10{}^{-9}$
		GMAD	4.860 × $10{}^{-8}$	2.960 × $10{}^{-8}$	3.280 × $10{}^{-8}$	7.390 × $10{}^{-9}$	5.080 × $10{}^{-7}$	3.040 × $10{}^{-7}$	1.470 × $10{}^{-7}$	1.160 × $10{}^{-7}$
3	E	GRMSE	6.120 × $10{}^{-8}$	3.870 × $10{}^{-8}$	4.380 × $10{}^{-8}$	8.780 × $10{}^{-9}$	6.210 × $10{}^{-7}$	3.760 × $10{}^{-7}$	1.820 × $10{}^{-7}$	1.460 × $10{}^{-7}$
		GENSE	1.450 × $10{}^{-12}$	1.090 × $10{}^{-11}$	1.830 × $10{}^{-13}$	6.480 × $10{}^{-13}$	5.900 × $10{}^{-9}$	2.930 × $10{}^{-9}$	1.330 × $10{}^{-9}$	5.760 × $10{}^{-9}$
		GMAD	3.900 × $10{}^{-8}$	4.330 × $10{}^{-8}$	2.830 × $10{}^{-8}$	1.350 × $10{}^{-8}$	3.170 × $10{}^{-7}$	2.010 × $10{}^{-7}$	1.990 × $10{}^{-7}$	1.530 × $10{}^{-7}$
	ϕ	GRMSE	5.420 × $10{}^{-8}$	4.770 × $10{}^{-8}$	3.390 × $10{}^{-8}$	1.520 × $10{}^{-8}$	3.920 × $10{}^{-7}$	2.510 × $10{}^{-7}$	2.500 × $10{}^{-7}$	1.950 × $10{}^{-7}$
		GENSE	1.330 × $10{}^{-12}$	1.210 × $10{}^{-13}$	9.560 × $10{}^{-12}$	2.040 × $10{}^{-13}$	5.280 × $10{}^{-9}$	3.610 × $10{}^{-9}$	1.540 × $10{}^{-8}$	3.680 × $10{}^{-8}$

## Data Availability

The data that support the findings of this study are available from the corresponding author upon reasonable request.

## Abbreviations

The following abbreviations are used in this manuscript:

MIN	Minimum
MAX	Maximum
DC	Direct Current
E	Electric Field
ρ	Charge Density
ϕ	Electric Potential
STD	Standard Devaition
SCA	Sine–Cosine Algorithm
RMSE	Root-Mean-Square Error
MAD	Mean Absolute Deviation
ANN	Artificial Neural Network
UP-EHD	Unipolar Electrohydrodynamic
ENSE	Error in Nash–Sutcliffe Efficiency
RK4	Runge–Kutta order four technique
SQP	Sequential Quadratic Programming

## Appendix A

Solution for problem 1 of UP-EHD is as follows: (subscripts c1, c2, c3, and c4 represent cases 1, 2, 3, and 4, respectively)

(A1) $$\begin{array}{c} \rho_{c1}\left(\eta \right)=\frac{0.686935018930724}{1+e^{-(-0.731321005263254\eta -1.60048418165935)}}+\frac{0.445130930819481}{1+e^{-(-0.160390042345572\eta +0.344966191572114)}}\\ +\frac{1.01775526912862}{1+e^{-(-0.237794215574325\eta +1.16424683215874)}}+\frac{0.295605759457221}{1+e^{-(0.398188964182966\eta -0.430424494985087)}}\\ +\frac{-0.541942801112263}{1+e^{-(0.0118850715177482\eta -0.0216778381698735)}},\\ E_{c1}\left(\eta \right)=\frac{1.47544444813911}{1+e^{-(0.421653523759878\eta -1.07416467420316)}}+\frac{2.34592147691180}{1+e^{-(0.665618324966216\eta +1.84398448894201)}}\\ +\frac{-3.34277783470822}{1+e^{-(-0.264899040759170\eta +0.422923845559382)}}+\frac{2.36014523365094}{1+e^{-(0.663867889065843\eta +1.79023864655830)}}\\ +\frac{-3.54158738023086}{1+e^{-(-0.380641059060822\eta +0.748314991050356)}},\\ \phi_{c1}\left(\eta \right)=\frac{-2.09269759226553}{1+e^{-(1.37560611768943\eta +0.793786737258395)}}+\frac{-0.781990818289106}{1+e^{-(-0.445855549657567\eta +0.129223385155023)}}\\ +\frac{0.901617278837918}{1+e^{-(-2.18106138933635\eta +2.33577562843877)}}+\frac{-2.42083352653851}{1+e^{-(-2.00300733008017\eta -1.47483938598153)}}\\ +\frac{2.50577589340382}{1+e^{-(-2.36248115538741\eta +4.83561987330236)}}. \end{array}$$

(A2) $$\begin{array}{c} \rho_{c2}\left(\eta \right)=\frac{0.403571230474982}{1+e^{-(0.0754773977304808\eta -0.953550172672349)}}+\frac{5.14629025112138}{1+e^{-(-8.35145964740123\eta -5.52180156863752)}}\\ +\frac{0.697467633381964}{1+e^{-(-0.432049911656944\eta +0.587443673696900)}}+\frac{1.61410447754806}{1+e^{-(-3.65783050693980\eta -2.33199238569362)}}\\ +\frac{0.881425405505853}{1+e^{-(-1.55641677113485\eta -0.785197500735136)}},\\ E_{c2}\left(\eta \right)=\frac{1.17826837259953}{1+e^{-(2.42754218903738\eta +1.92335459046939)}}+\frac{-3.12359013709183}{1+e^{-(-0.683688871596965\eta -0.292199027034798)}}\\ +\frac{1.34598947616311}{1+e^{-(0.428019979068469\eta -1.12456861423126)}}+\frac{-0.0463212432151125}{1+e^{-(1.13697716414593\eta -0.975925894711738)}}\\ +\frac{-4.07380545915162}{1+e^{-(-6.22604591692505\eta -5.99361250377857)}},\\ \phi_{c2}\left(\eta \right)=\frac{-3.84936904023960}{1+e^{-(0.392200191244766\eta +0.794797023955554)}}+\frac{-2.34633567943806}{1+e^{-(0.836806823545364\eta -0.0458076589687256)}}\\ +\frac{6.01003054174544}{1+e^{-(-0.940465537527358\eta +3.31458953216694)}}+\frac{-1.77052938445492}{1+e^{-(-1.37822972973792\eta -0.170286275399202)}}\\ +\frac{-4.21310762208361}{1+e^{-(-2.19056915117207\eta -3.04629044931212)}}. \end{array}$$

(A3) $$\begin{array}{c} \rho_{c3}\left(\eta \right)=\frac{0.627092471483705}{1+e^{-(-1.20859513123796\eta -0.0678985147351650)}}+\frac{0.772648139964832}{1+e^{-(-3.27939907023498\eta -0.897653328949199)}}\\ +\frac{8.36477219546771}{1+e^{-(-16.8661275829831\eta -5.39858893679546)}}+\frac{2.74673449770727}{1+e^{-(-7.08148858351234\eta -2.68781423232580)}}\\ +\frac{1.08534848776017}{1+e^{-(-0.0105948062713203\eta -1.15125364928352)}},\\ E_{c3}\left(\eta \right)=\frac{-1.16686229983182}{1+e^{-(-0.206737979771306\eta +0.518195926157895)}}+\frac{-1.36093852098816}{1+e^{-(-0.746189349453227\eta -0.459144018390082)}}\\ +\frac{0.756151814850389}{1+e^{-(0.816064568551740\eta -0.281497232270596)}}+\frac{-5.93949313415211}{1+e^{-(-9.87186250009969\eta -5.89883284559016)}}\\ +\frac{1.09766725431272}{1+e^{-(3.05007827471768\eta +1.85585966671120)}},\\ \phi_{c3}\left(\eta \right)=\frac{0.246053485292417}{1+e^{-(4.31330259430395\eta -8.29098565088038)}}+\frac{12.1151019962322}{1+e^{-(-0.246458663190581\eta -1.87440224280163)}}\\ +\frac{-11.7268930780487}{1+e^{-(-4.02421309061964\eta -6.21896607090309)}}+\frac{-5.97534820587807}{1+e^{-(-1.26067376494993\eta -2.69910587786410)}}\\ +\frac{-6.17110011180150}{1+e^{-(0.950674021005168\eta -3.33674729501039)}}. \end{array}$$

(A4) $$\begin{array}{c} \rho_{c4}\left(\eta \right)=\frac{-0.380195942387256}{1+e^{-(-0.339679496337086\eta +0.372826766781163)}}+\frac{7.92551764962122}{1+e^{-(-7.51543943479434\eta -3.21017886251748)}}\\ +\frac{-0.535343308994106}{1+e^{-(2.94151822873970\eta +0.216072953645222)}}+\frac{11.7165770451063}{1+e^{-(-23.5498963102440\eta -4.76268529213255)}}\\ +\frac{2.47880939715670}{1+e^{-(-0.177891804876677\eta -0.201470683944969)}},\\ E_{c4}\left(\eta \right)=\frac{-7.09901961859712}{1+e^{-(-22.0206212842505\eta -7.05352336080341)}}+\frac{-0.997502582490959}{1+e^{-(0.567552164735934\eta +0.345933757400632)}}\\ +\frac{0.679083998138482}{1+e^{-(1.45690537383910\eta -2.67616403763446)}}+\frac{-1.61158697479998}{1+e^{-(-5.39625252748116\eta -2.84615102747220)}}\\ +\frac{1.18711922553375}{1+e^{-(1.66534526395737\eta +0.139916439045306)}},\\ \phi_{c4}\left(\eta \right)=\frac{1.48497384437171}{1+e^{-(-1.48503296487957\eta +2.32849872690557)}}+\frac{-0.310155715544976}{1+e^{-(4.84780053053754\eta +1.55742012714371)}}\\ +\frac{2.77520626337140}{1+e^{-(-4.89422382430920\eta -1.52844540007968)}}+\frac{-1.48279788959264}{1+e^{-(-4.73420405647983\eta -1.15642603636405)}}\\ +\frac{-2.18686316575609}{1+e^{-(-5.23933151773142\eta -2.10908108823179)}}. \end{array}$$

Solutions of all cases of problem 2:

(A5) $$\begin{array}{c} \rho_{c1}\left(\eta \right)=\frac{-0.224444075499975}{1+e^{-(-3.63139884645821\eta -0.920646415824820)}}+\frac{1.43925810926544}{1+e^{-(-0.591881813143987\eta -1.10920216618766)}}\\ +\frac{4.14831734559800}{1+e^{-(-3.99152465315082\eta -2.46013483041525)}}+\frac{1.20604748772851}{1+e^{-(-1.79190787664097\eta -0.911496779429942)}}\\ +\frac{5.85676433186023}{1+e^{-(-10.5637488947129\eta -5.12203366802729)}},\\ E_{c1}\left(\eta \right)=\frac{-0.701753832816577}{1+e^{-(-0.316732732393901\eta -0.332294829200489)}}+\frac{-1.72261842238091}{1+e^{-(-2.46545311738499\eta -1.87420086304847)}}\\ +\frac{-1.79210515209530}{1+e^{-(-0.827548660875994\eta -0.456112895006224)}}+\frac{1.60515331801270}{1+e^{-(6.83962915349901\eta +4.23371761571914)}}\\ +\frac{-1.14166108137094}{1+e^{-(0.204588331776970\eta -0.756212300809000)}},\\ \phi_{c1}\left(\eta \right)=\frac{-2.12760603867376}{1+e^{-(1.15275152907962\eta -1.95992049891398)}}+\frac{0.664269584529447}{1+e^{-(3.65535701279987\eta +2.70305254275597)}}\\ +\frac{-2.15886937281600}{1+e^{-(-1.98026820632127\eta -3.19999172986440)}}+\frac{0.399868902002650}{1+e^{-(-0.289201404539833\eta +0.944327922815873)}}\\ +\frac{3.56781880484951}{1+e^{-(-0.0394840025338410\eta -1.96961472122974)}}. \end{array}$$

(A6) $$\begin{array}{c} \rho_{c2}\left(\eta \right)=\frac{0.291618330496683}{1+e^{-(-4.19313683045191\eta -0.517132604632150)}}+\frac{1.00771797946221}{1+e^{-(-1.74089694581232\eta -0.741643381846177)}}\\ +\frac{4.47724784124786}{1+e^{-(-7.07117166249586\eta -3.98378430492696)}}+\frac{0.264577911780102}{1+e^{-(-0.442123245005100\eta +0.111923031474055)}}\\ +\frac{0.730829511377141}{1+e^{-(-0.365871433970872\eta -0.114964465405928)}},\\ E_{c2}\left(\eta \right)=\frac{-0.244379384154477}{1+e^{-(-0.0308738816385429\eta +0.00229195946579636)}}+\frac{-3.77761742824242}{1+e^{-(-6.19180298993945\eta -5.44859579364093)}}\\ +\frac{1.31182386084363}{1+e^{-(0.961413400154481\eta -0.888942410945240)}}+\frac{0.324702928383232}{1+e^{0.309996386602437\eta -0.167820971522187)}}\\ +\frac{-3.24623424402549}{1+e^{-1.77686198360289\eta -1.98408115997852)}},\\ \phi_{c2}\left(\eta \right)=\frac{0.223526067359304}{1+e^{-(-2.92803014703422\eta -1.11157919362991)}}+\frac{-3.32005435544189}{1+e^{-(0.416548574581639\eta -0.733003375588457)}}\\ +\frac{-2.43898340388030}{1+e^{-(-2.18000124832846\eta -2.50556918365034)}}+\frac{2.64593868832854}{1+e^{-(-1.02865761692884\eta +2.39682249599780)}}\\ +\frac{-1.68054116764700}{1+e^{-(-1.37657227216077\eta -1.89805615783556)}}. \end{array}$$

(A7) $$\begin{array}{c} \rho_{c3}\left(\eta \right)=\frac{0.394075901193115}{1+e^{-(-4.40863417631040\eta -1.58164028077953)}}+\frac{4.83288601855934}{1+e^{-(-7.40719221930165\eta -4.80766625024176)}}\\ +\frac{0.827351826673359}{1+e^{-(-1.00930994535225\eta +0.222368824338441)}}+\frac{1.41586325451351}{1+e^{-(0.0906223018451300\eta +0.172875291151252)}}\\ +\frac{-0.505935206777341}{1+e^{-(2.75685642962720\eta +0.671244426210105)}},\\ E_{c3}\left(\eta \right)=\frac{-0.0668901799101151}{1+e^{-(0.979404034379458\eta +0.399736759343759)}}+\frac{-2.60932125180425}{1+e^{-(-1.35712134267543\eta -1.30203208161336)}}\\ +\frac{1.51355246416335}{1+e^{-(0.840321229988548\eta -1.28789687225012)}}+\frac{1.43072985477591}{1+e^{0.222364021269655\eta -1.30557066055915)}}\\ +\frac{-3.30679805524652}{1+e^{-4.45805769512058\eta -4.55692615019554)}},\\ \phi_{c3}\left(\eta \right)=\frac{-2.74358896061384}{1+e^{-(1.37341145674806\eta -3.04997436807209)}}+\frac{0.650816481057709}{1+e^{-(-1.62539127856035\eta +1.80425438167343)}}\\ +\frac{-0.623837731435995}{1+e^{-(-0.0600644882348463\eta +0.163810980819659)}}+\frac{0.0542058077588113}{1+e^{-(0.773636909854753\eta +0.208017054629008)}}\\ +\frac{0.975516267808246}{1+e^{-(2.94977120835981\eta +2.13891605474960)}}. \end{array}$$

(A8) $$\begin{array}{c} \rho_{c4}\left(\eta \right)=\frac{0.403571230474982}{1+e^{-(0.0754773977304808\eta -0.953550172672349)}}+\frac{5.14629025112138}{1+e^{-(-8.35145964740123\eta -5.52180156863752)}}\\ +\frac{0.697467633381964}{1+e^{-(-0.432049911656944\eta +0.587443673696900)}}+\frac{1.61410447754806}{1+e^{-(-3.65783050693980\eta -2.33199238569362)}}\\ +\frac{0.881425405505853}{1+e^{-(-1.55641677113485\eta -0.785197500735136)}},\\ E_{c4}\left(\eta \right)=\frac{1.17826837259953}{1+e^{-(2.42754218903738\eta +1.92335459046939)}}+\frac{-3.12359013709183}{1+e^{-(-0.683688871596965\eta -0.292199027034798)}}\\ +\frac{1.34598947616311}{1+e^{-(0.428019979068469\eta -1.12456861423126)}}+\frac{-0.0463212432151125}{1+e^{1.13697716414593\eta -0.975925894711738)}}\\ +\frac{-4.07380545915162}{1+e^{-6.22604591692505\eta -5.99361250377857)}},\\ \phi_{c4}\left(\eta \right)=\frac{-3.84936904023960}{1+e^{-(0.392200191244766\eta +0.794797023955554)}}+\frac{-2.34633567943806}{1+e^{-(0.836806823545364\eta -0.0458076589687256)}}\\ +\frac{6.01003054174544}{1+e^{-(-0.940465537527358\eta +3.31458953216694)}}+\frac{-1.77052938445492}{1+e^{-(-1.37822972973792\eta -0.170286275399202)}}\\ +\frac{-4.21310762208361}{1+e^{-(-2.19056915117207\eta -3.04629044931212)}}. \end{array}$$

Solution of all cases of problem 3:

(A9) $$\begin{array}{c} \rho_{c1}\left(\eta \right)=\frac{0.394075901193115}{1+e^{-(-4.40863417631040\eta -1.58164028077953)}}+\frac{4.83288601855934}{1+e^{-(-7.40719221930165\eta -4.80766625024176)}}\\ +\frac{0.827351826673359}{1+e^{-(-1.00930994535225\eta +0.222368824338441)}}+\frac{1.41586325451351}{1+e^{-(0.0906223018451300\eta +0.172875291151252)}}\\ +\frac{-0.505935206777341}{1+e^{-(2.75685642962720\eta +0.671244426210105)}},\\ E_{c1}\left(\eta \right)=\frac{-0.0668901799101151}{1+e^{-(0.979404034379458\eta +0.399736759343759)}}+\frac{-2.60932125180425}{1+e^{-(-1.35712134267543\eta -1.30203208161336)}}\\ +\frac{1.51355246416335}{1+e^{-(0.840321229988548\eta -1.28789687225012)}}+\frac{1.43072985477591}{1+e^{0.222364021269655\eta -1.30557066055915)}}\\ +\frac{-3.30679805524652}{1+e^{-4.45805769512058\eta -4.55692615019554)}},\\ \phi_{c1}\left(\eta \right)=\frac{-2.74358896061384}{1+e^{-(1.37341145674806\eta -3.04997436807209)}}+\frac{0.650816481057709}{1+e^{-1.62539127856035\eta +1.80425438167343)}}\\ +\frac{-0.623837731435995}{1+e^{-(-0.0600644882348463\eta +0.163810980819659)}}+\frac{0.0542058077588113}{1+e^{-(0.773636909854753\eta +0.208017054629008)}}\\ +\frac{0.975516267808246}{1+e^{-(2.94977120835981\eta +2.13891605474960)}}. \end{array}$$

(A10) $$\begin{array}{c} \rho_{c2}\left(\eta \right)=\frac{0.634325052987188}{1+e^{-(-2.33077777766693\eta -1.04154448976362)}}+\frac{0.779518982315070}{1+e^{-(-0.0287302281367053\eta -0.163110433555730)}}\\ +\frac{1.99914299825649}{1+e^{-(-0.854796393084468\eta -2.56319745913218)}}+\frac{0.554219882721009}{1+e^{-(-0.695213550093129\eta +0.0200977826398004)}}\\ +\frac{3.43298921049676}{1+e^{-(-5.11076050851875\eta -4.14449337088888)}},\\ E_{c2}\left(\eta \right)=\frac{0.979037616331823}{1+e^{-(0.564485797455951\eta -0.877466243209346)}}+\frac{-2.84447545474630}{1+e^{-(-3.39397988524584\eta -4.16697081776192)}}\\ +\frac{0.516137618484723}{1+e^{-(0.860384171322143\eta +0.290273259562283)}}+\frac{-1.44176752479047}{1+e^{-0.472617934296873\eta +0.691699283066018)}}\\ +\frac{0.623745836339739}{1+e^{1.68251400400149\eta +0.733010876039821)}},\\ \phi_{c2}\left(\eta \right)=\frac{0.719887961522569}{1+e^{-(2.94036576440455\eta +2.43443372771069)}}+\frac{-3.08249113218188}{1+e^{1.22182052637383\eta -2.71182472345210)}}\\ +\frac{0.513860049679870}{1+e^{-(1.11772026353975\eta +1.00525085724163)}}+\frac{0.159381958622984}{1+e^{-(-2.08775677861021\eta +1.50198680899259)}}\\ +\frac{0.0967513386193249}{1+e^{-(0.000429417602955564\eta -1.13396203042176)}}. \end{array}$$

(A11) $$\begin{array}{c} \rho_{c3}\left(\eta \right)=\frac{0.00330275564797704}{1+e^{-(-3.50196533815681\eta +1.28058309998887)}}+\frac{1.67188074839827}{1+e^{-(-3.73475512018102\eta -3.71574290392104)}}\\ +\frac{1.91732484841867}{1+e^{-(-1.28670103431850\eta -1.76178325988994)}}+\frac{0.490832293835132}{1+e^{-(-0.377255933479913\eta +1.51305926612333)}}\\ +\frac{0.537538357444175}{1+e^{-(-0.276887697407495\eta +0.0421451720697853)}},\\ E_{c3}\left(\eta \right)=\frac{2.32854831032098}{1+e^{-(-1.36343825671273\eta -0.0644549370939950)}}+\frac{-3.30820487352466}{1+e^{-(-0.931579981217383\eta +0.0726746113863261)}}\\ +\frac{0.977535350204778}{1+e^{-(1.73106037511433\eta +0.285203391714108)}}+\frac{0.0990297429759537}{1+e^{-(1.61758180348554\eta +0.0494383415725308)}}\\ +\frac{-3.73646328242681}{1+e^{-3.24898446932002\eta -5.16045993118277)}},\\ \phi_{c3}\left(\eta \right)=\frac{-2.90934003752946}{1+e^{-(-0.884450134602017\eta -1.57633696961055)}}+\frac{2.48138864006450}{1+e^{-0.158746931722210\eta +2.29906956296800)}}\\ +\frac{-4.38896544332240}{1+e^{-(0.404702672673122\eta +0.246393450361488)}}+\frac{1.93198998492294}{1+e^{-(-1.12736480988621\eta +3.20833163738272)}}\\ +\frac{-2.71241428699855}{1+e^{-(-1.31151901735734\eta -2.83722421856703)}}. \end{array}$$

(A12) $$\begin{array}{c} \rho_{c4}\left(\eta \right)=\frac{0.946431643600176}{1+e^{-(-2.16498871519545\eta -3.11259053166308)}}+\frac{0.605637726668847}{1+e^{-(-0.239431825840412\eta +0.145252340769612)}}\\ +\frac{0.922557761443514}{1+e^{-(-0.00803237451753994\eta -0.870451722830120)}}+\frac{-0.418513853057636}{1+e^{-(-0.991770096015404\eta +1.12898455171492)}}\\ +\frac{1.39165986729996}{1+e^{-(-0.648446844719879\eta -0.0487668410331998)}}\\ E_{c4}\left(\eta \right)=\frac{-2.24774344025241}{1+e^{-(-1.27939983522288\eta -3.35361184748167)}}+\frac{0.977568103018951}{1+e^{-(-1.26224495048768\eta +0.225726190411781)}}\\ +\frac{-0.722698267384792}{1+e^{-(0.751439944062959\eta +0.320704902056278)}}+\frac{1.45638924095064}{1+e^{-(1.21469328850630\eta -0.0611890030806621)}}\\ +\frac{-1.02123719483070}{1+e^{-0.768057329031559\eta +1.04190848041238)}}\\ \phi_{c4}\left(\eta \right)=\frac{0.626219343287679}{1+e^{-(1.74610644064281\eta +2.06928126000693)}}+\frac{1.35861645555303}{1+e^{-(-1.10889337652674\eta +2.45822514966068)}}\\ +\frac{-0.515842907760147}{1+e^{-(0.153195186149203\eta +0.0784579039446797)}}+\frac{-0.527608291658212}{1+e^{-(-0.118737764128748\eta -0.267669735053490)}}\\ +\frac{-0.576702496454243}{1+e^{-(-0.0342677365461796\eta +0.155816426372289)}}. \end{array}$$
